# How Cross-Cue Inconsistency Weakens the Adoption of AI-Generated Review Summaries: The Roles of Diagnosticity, Authenticity, and Review Dispersion

**DOI:** 10.3390/bs16091516

**Published:** 2026-08-28

**Authors:** Wei He, Hua Meng, Xinyuan Lu

**Affiliations:** 1 School of Information Management, Central China Normal University, Wuhan 430079, China; wei.he@mails.ccnu.edu.cn (W.H.); luxy@mail.ccnu.edu.cn (X.L.); 2 School of Information Management, Wuhan University, Wuhan 430072, China; 3 Hubei E-Commerce Research Center, Central China Normal University, Wuhan 430079, China

**Keywords:** AI-generated summary (AIGS), aggregated user-generated rating (AUGR), AIGS-AUGR inconsistency, perceived diagnosticity, perceived authenticity, review dispersion, adoption intention

## Abstract

As AI-generated review summaries are increasingly embedded in e-commerce review systems, consumers are no longer exposed to a single type of user review information. Instead, they encounter multiple evaluative cues consisting of aggregated user-generated ratings (AUGR), original user reviews, and AI-generated summaries (AIGS). From the perspective of cross-cue inconsistency, this study examines the mechanism underlying consumers’ adoption of AIGS in a complex evaluative environment. This study adopts a scenario-based experimental method and simulates a product review page on an e-commerce platform. A 2 (AIGS: positive vs. negative) × 2 (AUGR: high vs. low) × 2 (review dispersion: low vs. high) between-subjects experimental design was employed. Data from 371 participants from mainland China recruited through Credamo online panel were analyzed using full factorial ANOVA and PROCESS-based mediation and moderated mediation analyses. AIGS-AUGR consistency/inconsistency was generated by combining the valence of AIGS with the level of AUGR, thereby capturing different manifestations of cross-cue consistency/inconsistency. Based on this design, consumers’ perceived diagnosticity, perceived authenticity, and adoption intention were measured, and the proposed hypotheses were tested. The results show that AIGS-AUGR inconsistency significantly reduces consumers’ adoption intention toward AIGS. In the overall mediation analysis, perceived diagnosticity and perceived authenticity both mediate this effect. Review dispersion differentially moderates the first-stage effects of inconsistency on the two mediators: high review dispersion strengthens its negative effect on perceived diagnosticity, whereas low review dispersion strengthens its negative effect on perceived authenticity. However, only the diagnosticity-based indirect effect on adoption intention varies significantly across dispersion levels, with high review dispersion strengthening this negative indirect effect; the moderated indirect effect through perceived authenticity is not significant.

## 1. Introduction

As the volume of reviews on e-commerce platforms continues to grow, online reviews have become an important information source for consumers to understand product quality and reduce transaction uncertainty. Recent studies have shown that specific textual characteristics of online reviews, such as review content and sentiment expressions, influence consumers’ evaluations and subsequent market outcomes (Zhai et al., 2024). At the same time, however, the expansion of review volume and format has created new pressures for information processing. Some studies have suggested that while a large number of reviews can provide rich information, they may also lead to comprehension difficulties and information overload (Jabr & Rahman, 2022). As a result, platforms often extract key signals through mechanisms such as selected reviews. With the increasing presence of multimodal reviews, including images and videos, the complexity of review content has further increased. Excessive information may exceed users’ cognitive processing capacity and thereby reduce decision quality (Qiao et al., 2025). Against this background, generative artificial intelligence (GenAI) has begun to be applied to online review systems. Existing research suggests that generative AI reviews, as a novel form of online evaluation, can extract commonalities and differences from large volumes of reviews and provide consumers with more concise product information (Lei & Liu, 2025). Platforms such as Amazon and Newegg are adopting AI-generated summaries (AIGS) to reduce consumers’ reading costs and help them obtain review information more efficiently (Z. Li et al., 2026). Therefore, AIGS has become an important phenomenon in the evolution of online review systems and has raised new research questions about how consumers use platform-generated evaluative information.

However, AIGS does not operate independently of the original review system. On review pages, consumers are usually exposed simultaneously to aggregated user-generated ratings (AUGR), individual reviews, review sentiment, platform labels, and summary information, and they compare these cues with one another. Existing research has shown that inconsistency between easy-to-retrieve review ratings and aggregate ratings can influence the cognitive heuristics consumers rely on when interpreting online reviews (R. Zhang et al., 2024). A mismatch between ratings and textual content can also change perceived review usefulness, leading consumers to reassess whether a review is worth using as a reference (Kwon et al., 2025). In the context of restaurant reviews, inconsistency among ratings, service dimensions, and textual evaluations likewise affects consumers’ judgments of review helpfulness (Park & Park, 2025). Related studies have also found that inconsistency between consumer reviews and management responses can reduce the helpfulness of review sets and further influence business outcomes (P. Jiang & Fan, 2026). In addition, inconsistency between influencer-generated content and user-generated content (UGC) can also shape consumers’ judgments of information authenticity and persuasiveness (Y. Liu et al., 2025b). Taken together, these findings suggest that in environments where multiple evaluative cues coexist, consumers do not focus only on a single piece of information itself; rather, they also attend to whether different pieces of information are mutually consistent. Therefore, when inconsistency arises between AIGS and user review information, the function originally intended to reduce information processing costs may instead become a new source of judgment difficulty.

Existing research has provided a foundation for understanding AI-generated evaluative information, but several issues remain unresolved. Prior studies have shown that GenAI is reshaping how consumers search, receive, and evaluate information by shifting AI from prediction-oriented tools toward content-generation systems (Hermann & Puntoni, 2024). Research in online review and content-generation contexts has further indicated that consumers consider factors such as usefulness, credibility, and authenticity when evaluating AI-generated information (Amos & Zhang, 2024; Belanche et al., 2025). However, existing studies have primarily examined consumers’ responses to AI-generated content itself or compared AI-generated content with human-generated content, while paying limited attention to how AIGS interacts with other evaluative cues already embedded in review systems. In particular, it remains unclear why consumers reduce their adoption intention when AIGS conflicts with AUGR, and whether characteristics of original reviews, such as review dispersion, influence this process. Therefore, this study examines the psychological mechanisms and boundary conditions underlying consumers’ adoption of AIGS in multi-cue review environments.

Accordingly, this study shifts attention from whether AI-generated summaries are generally useful to when and why consumers accept or reject them in relation to other evaluative cues. This study focuses on three research questions:

- RQ1. Does AIGS-AUGR inconsistency reduce consumers’ adoption intention toward AIGS?
- RQ2. Does this effect occur through two pathways, namely perceived diagnosticity and perceived authenticity? In other words, do consumers reduce their adoption because they perceive the summary as not useful enough, or because they believe that it fails to authentically reflect user reviews?
- RQ3. Does review dispersion change this process? Specifically, do consumers interpret AIGS-AUGR inconsistency differently when review opinions are highly concentrated versus clearly divided? More specifically, this study examines whether high review dispersion makes cross-cue inconsistency more consequential for perceived diagnosticity, whereas low review dispersion makes it more consequential for perceived authenticity.

To answer these questions, this study adopts a scenario-based experimental method and simulates a product review interface on an e-commerce platform. It examines AIGS-AUGR consistency/inconsistency and review dispersion, and tests their effects on consumers’ psychological responses and adoption intention. The contribution of this study lies in examining AIGS within a multi-cue evaluative environment, distinguishing between the diagnosticity and authenticity pathways, and examining how review dispersion differentially shapes consumers’ interpretations of cross-cue inconsistency. The findings also provide practical implications for platforms to optimize the generation and presentation of AIGS.

## 2. Literature Review

### 2.1. Evolution of Online Review Systems

Early online review systems mainly presented original reviews in a linear format. Consumers usually had to read review texts one by one to form a basic judgment about a product or service. As the volume of reviews increased rapidly, platforms were no longer satisfied with merely storing reviews; instead, they began to organize review information in a more layered manner (Qiu & Zhang, 2024). Research has shown that, in many contexts, aggregated textual reviews are more likely to be perceived as useful by consumers than individual reviews (Jin et al., 2023). This indicates that review systems have evolved from a single content-display format to a presentation structure in which overall evaluations and individual evaluations coexist.

In this process, the interface structure and ranking rules of review systems have also become increasingly important. Platforms do not present reviews in a neutral manner; rather, they shape consumers’ information exposure through ranking mechanisms and positional allocation. The visibility of reviews in rankings such as most helpful or most recent significantly affects their chances of being read and further used (Alzate et al., 2024). The presentation of reviews has also gradually expanded from plain text to multimodal forms that combine text and images (Pocchiari et al., 2025). Studies have shown that text–image multimodal reviews influence consumers’ perceptions of review usefulness, while sensory differences between modalities may weaken this effect (S. Sun et al., 2025).

GenAI has further promoted the evolution of review systems. AIGS has been embedded in the review interfaces of multiple e-commerce platforms. It is usually placed before original reviews and is used to extract key information from large volumes of user reviews, thereby alleviating information overload and improving the usability of review sections (M. Jia et al., 2026). In this new structure, platforms often present AUGR and AIGS simultaneously, and these two cues jointly serve as key entry points for consumers to understand overall evaluations (L. Luo et al., 2026). Thus, online review systems have gradually evolved into composite information interfaces in which ratings, rankings, individual reviews, multimodal content, and AIGS jointly shape consumers’ initial product judgments. However, existing research has mainly examined how these components individually influence information processing, while paying less attention to how consumers respond when different evaluative cues provided by the platform and users are inconsistent.

### 2.2. Consumer Judgment Mechanisms Under Multiple Evaluative Cues

In the context of online reviews, consumers usually do not rely on a single cue when making judgments. Instead, within a limited amount of time, they integrate multiple types of information, such as ratings, review text, review length, reviewer identity, images, and platform labels, to form an overall perception of both the product and the review itself (S. Sun et al., 2025). Recent studies generally regard online review judgment as a process of multi-cue integration, in which different cues play different roles in information processing (Mousavizadeh et al., 2020). Under conditions of limited attention, consumers may make quick judgments based on some cues, while engaging in deeper processing of cues with higher diagnostic value (Kakaria et al., 2023; Rosillo-Díaz et al., 2024).

From the perspective of content, review text remains an important basis for consumers’ in-depth judgment. Studies have shown that review readability, length, sentiment valence, and information completeness significantly affect review helpfulness and use value (J. Liu et al., 2025). In particular, greater textual clarity and broader information coverage usually help consumers better understand product attributes and reduce search costs (X. Sun et al., 2019). At the same time, quantitative cues such as ratings often have strong heuristic effects and can help consumers quickly form initial impressions (R. Zhang et al., 2024). Therefore, when time is limited or cognitive effort is low, consumers are more likely to rely first on such simplified signals. In addition to text and ratings, peripheral cues in review systems can also influence consumer judgments (J. Liu et al., 2025; L. Luo et al., 2024). Evidence from Amazon reviews indicates that verified purchase badges and user-uploaded images enhance review helpfulness, and that the positive effect becomes stronger as the number of images increases (H. S. Choi & Leon, 2025). Further evidence suggests that review images are not always effective; their effects depend on factors such as product type, review length, and reviewer reputation (Kübler et al., 2024). This suggests that consumers do not mechanically accept visual cues, but evaluate their diagnostic value in relation to specific contexts.

However, multiple evaluative cues do not simply have additive effects; rather, they often interact with one another. Evidence from multimodal review research indicates that high-quality text and high-quality images jointly improve review usefulness, and that their combined effect is stronger than the independent effect of either cue alone (Y. Zhang et al., 2025). Meanwhile, some cues that appear to help build trust may also produce the opposite effect. For example, research has shown that review verification labels may, in some contexts, trigger consumers’ suspicion of manipulation and thereby weaken review credibility (Mardumyan & Siret, 2023). Therefore, consumer judgment in a multi-cue environment is not a static process. Instead, consumers interpret review information by considering the degree of fit among cues, the meaning of the specific context, and the structure of the platform interface. Nevertheless, existing multi-cue studies have primarily focused on the additive or complementary effects of different information cues, leaving insufficient explanation of the negative consequences when AI-generated and user-generated cues conflict with each other.

### 2.3. Evaluation Conflict, Information Incoordination, and Cross-Cue Inconsistency

In online review research, evaluation conflict has long been an important issue for understanding consumers’ information processing. Earlier studies often conceptualized such conflict as review disagreement or rating dispersion. For example, prior evidence suggests that higher review variance increases consumers’ decision uncertainty and reduces purchase intention (Langan et al., 2017). Related research further indicates that rating dispersion changes consumers’ trust in average ratings and their motivation to read individual reviews (S. Lee et al., 2021). More recent studies further suggest that consumers are not usually faced with isolated individual reviews, but rather with a review set composed of multiple representative reviews (Wan & Sam, 2024). In this context, the concept of cross-review incoherence has been introduced to describe mutually contradictory information provided by different reviewers regarding the same product attribute. Empirical findings show that such incoherence reduces consumers’ evaluations of the helpfulness and credibility of a review set, thereby increasing their tendency to defer purchase decisions (Yin et al., 2023). At the same time, this negative effect can be weakened when reviewers provide more sufficient contextual information to explain their evaluative views. This indicates that when consumers can understand why different evaluative cues diverge, the negative impact of information incoordination can be partially alleviated.

In addition to disagreement across reviews, researchers have also begun to examine incoordination within individual reviews and across different evaluative cues. Evaluation conflict has also been distinguished into internal and external inconsistencies. The former mainly refers to contradictory statements within the same review, whereas the latter refers to an obvious deviation between a given review and other reviews. Evidence indicates that internal inconsistency is more likely to cause comprehension difficulty and thus reduce review helpfulness, while the effect of external inconsistency is more complex and depends on factors such as review length and the comparison context (J. Choi et al., 2023). Similarly, from the perspective of the relationship between content and context, topic deviations between a review and the product description or existing reviews have been shown to reduce review helpfulness (S. Wang et al., 2025). This suggests that consumers evaluate the value of a review by placing it within the broader information context rather than interpreting it in isolation. Therefore, consumers’ judgments of review information depend not only on a single cue itself, but also on whether that cue forms a coordinated relationship with other evaluative cues.

At present, whether ratings are consistent with textual sentiment has also become an important direction in evaluation conflict research. Evidence from rating–text consistency research suggests that star ratings and review sentiment are generally aligned, but clear rating–text mismatches also exist in mixed-neutral reviews (Bigne et al., 2023). This means that numerical ratings do not always accurately represent the content of reviews. When high ratings are accompanied by negative text, or when low ratings are accompanied by positive text, consumers are more likely to experience confusion and reassess the authenticity and diagnostic value of the review (P. Wang et al., 2025). The complementary roles of review sentiment and star ratings in shaping product demand have also been documented, suggesting that once the two lose their fit, consumers’ interpretation process may be affected (Cho et al., 2022). Existing studies have provided evidence that evaluation conflict can influence how consumers understand, trust, and use review information. However, prior research has mainly focused on inconsistency within user reviews, across reviews, or between ratings and text, while paying insufficient attention to cross-cue inconsistency between AIGS as a platform-generated cue and AUGR as a user-generated cue. This limitation becomes increasingly important as platforms introduce AI-generated summaries into existing review systems, because consumers must evaluate not only whether individual cues are credible but also whether different sources of evaluation are mutually aligned.

From this perspective, AIGS-AUGR inconsistency can be understood as an extension of external inconsistency rather than an entirely new form of inconsistency. Similar to prior research on external inconsistency, it reflects a mismatch between one evaluative cue and another source of information within the broader review environment (J. Choi et al., 2023). However, it differs from previous forms of inconsistency because the conflicting cues originate from different sources and represent different levels of evaluation: AIGS is a platform-generated summary derived from review content, whereas AUGR represents aggregated user-generated evaluations. Therefore, this study conceptualizes AIGS-AUGR inconsistency as a cross-source and cross-level external inconsistency that emerges when AI-generated summaries fail to align with existing user-generated evaluation cues.

### 2.4. Acceptance of AI-Generated Evaluative Information

Research on AI disclosure and authenticity has shown that AI labels can influence consumer attitudes and behavioral responses (Drozd & Söilen, 2026). Research has shown that AI disclosure can change consumers’ evaluations of advertising content (Baek et al., 2024). Some studies have also indicated that AIGC may affect consumer evaluations due to insufficient authenticity (G. Lee & Kim, 2024; J. Luo et al., 2026). However, these studies have not fully answered a more specific question: when AIGS is inconsistent with AUGR, why do consumers reduce their adoption of the summary, and does this effect depend on the degree of dispersion in the original reviews? Accordingly, this study further examines these issues from the perspectives of perceived diagnosticity, perceived authenticity, and review dispersion.

As GenAI enters e-commerce and platform contexts, the sources of evaluative information are changing. In the past, consumers were mainly exposed to reviews written by users themselves. Today, platforms have begun to provide AI-generated reviews, AIGS, and AI-integrated word-of-mouth information (M. Jia et al., 2026; Ma et al., 2026). Accordingly, related research has gradually shifted from how consumers use user reviews to how consumers understand, accept, and use machine-generated evaluative information (Cheng et al., 2025; M. Jia et al., 2026). Recent studies suggest that one important value of AI-generated evaluative information lies in reducing information overload and lowering reading costs (Y. Wang et al., 2026). However, such convenience does not automatically lead to adoption, as users often further assess whether the information is authentic, reliable, and suitable for the current decision context (S. J. Jia et al., 2025; M. Jia et al., 2026).

In terms of research themes, the first stream of recent literature mainly compares AI-generated evaluative information with human-generated evaluative information. Evidence from a comparison of AI-generated and human-generated reviews suggests that consumers may distinguish between the two types of content and adjust their usage tendencies accordingly (Cheng et al., 2025). This indicates that, in evaluative information contexts, who generates the information has itself become an important judgment cue. A related stream of research on brand content further suggests that GenAI disclosure may weaken users’ perceived authenticity of content and influence their behavioral responses (Brüns & Meißner, 2024). These findings suggest that once users become aware that information is generated by AI, their judgment criteria tend to change. Related research further suggests that such changes in judgment are closely associated with perceived authenticity and credibility. For example, Baek et al. (2024) found that AI disclosure influences consumers’ evaluations of advertising content by affecting their perceptions of authenticity and credibility. Related evidence similarly suggests that perceived authenticity plays an important role in shaping consumer responses to AIGC (G. Lee & Kim, 2024). The second stream of literature focuses more on why AI-generated evaluative information is accepted. Prior work on AIGC adoption indicates that both content cues and interaction cues affect users’ acceptance of AIGC (C. Li et al., 2025). In addition, authenticity perceptions have been identified as an important mechanism underlying users’ responses to AI-mediated information. Further research indicates that perceived authenticity links AI-related cues with consumer trust, suggesting that users’ acceptance of AI-generated information depends not only on functional benefits but also on whether such information is perceived as genuine and faithful (Drozd & Söilen, 2026). Users do not evaluate only the output itself, but also attend to how the content is presented, the sense of interaction, and their subjective experience during use. Extending this logic to the review context, recent findings suggest that the comprehensibility, transparency, and convenience of AIGS influence users’ credibility judgments, and that perceived credibility subsequently affects perceived helpfulness and adoption intention (M. Jia et al., 2026). Therefore, the use of AI-generated evaluative information is not merely a matter of efficiency, but also a process of cognitive judgment and trust formation. Taken together, existing research suggests that consumers’ acceptance of AI-generated evaluative information depends not only on its functional value but also on whether it is perceived as a credible and faithful representation of the underlying information. However, limited attention has been paid to how these judgments change when AI-generated summaries conflict with other evaluative cues presented in the same review environment.

## 3. Hypothesis Development

This study adopts cue utilization theory as its overarching theoretical framework. The integration of these perspectives follows from the nature of consumer evaluation in a multi-cue review environment. Cue utilization theory explains the initial comparison process through which consumers jointly interpret AIGS, AUGR, and original reviews and recognize whether these evaluative cues are mutually consistent. Once inconsistency is detected, consumers must evaluate both whether the AIGS remains useful for making product judgments and whether it faithfully represents the underlying user evaluations. Accessibility–diagnosticity theory explains the former functional judgment, whereas the attribution-based authenticity logic explains the latter representational judgment. Together, these perspectives connect cross-cue comparison with two distinct but complementary evaluations that shape consumers’ adoption of AIGS.

In the online review environment, AIGS, AUGR, and original user reviews constitute interrelated evaluative cues, and consumers assess AIGS not only on the basis of its standalone content but also by comparing it with other available cues. Cue utilization theory therefore explains why consistency or inconsistency among these cues shapes consumers’ overall evaluations of AIGS (Kakaria et al., 2023). Within this overarching framework, accessibility–diagnosticity theory specifies the diagnosticity pathway: cross-cue inconsistency may reduce the extent to which AIGS is perceived as useful for understanding product information and supporting judgment (Chen et al., 2024). In parallel, an attribution-based authenticity logic explains the authenticity pathway: when AIGS diverges from user-generated evaluative cues, consumers may attribute the discrepancy to selective, distorted, or insufficiently faithful summarization, thereby lowering perceived authenticity (Brüns & Meißner, 2024; Kirk & Givi, 2025). Thus, cue utilization theory provides the integrative foundation of the model, whereas accessibility–diagnosticity theory and authenticity attribution specify the two psychological mechanisms through which cross-cue inconsistency influences adoption intention. Review dispersion further shapes the informational context in which these two implications of inconsistency are interpreted.

### 3.1. The Effect of AIGS-AUGR Inconsistency on Adoption Intention

Consistent with cue utilization theory, consumers in online review environments do not interpret AIGS in isolation. Instead, they integrate AIGS with evaluative cues such as AUGR and original user reviews to form an overall judgment (J. Choi et al., 2023). Prior research has shown that platforms have begun to embed AIGS into review interfaces to help consumers quickly grasp key review information (M. Jia et al., 2026). However, whether users are willing to use such summaries largely depends on whether they are consistent with other evaluative information (L. Luo et al., 2026).

When AIGS is consistent with AUGR, consumers are more likely to regard it as an effective condensation of original reviews. As a result, they may perceive these summaries as helping them understand product evaluations more quickly and supporting subsequent judgment (M. Jia et al., 2026; Y. Wang et al., 2026). By contrast, when AIGS is inconsistent with AUGR or with the overall tendency of user reviews, consumers are more likely to believe that the summarized information provided by the platform fails to accurately reflect authentic user evaluations, thereby reducing their positive evaluation of the summary. Recent work has shown that sentiment mismatch between AIGS and AUGR weakens user attitudes (L. Luo et al., 2026). Meanwhile, research on traditional review systems has also shown that evaluation conflict and external inconsistency usually reduce review helpfulness (J. Choi et al., 2023; P. Wang et al., 2025) and hinder consumers from forming stable judgments (Yin et al., 2023).

In addition, in the context of AI-generated evaluative information, consumers tend to be more cautious than they are when facing ordinary user reviews (Cheng et al., 2025). Compared with human-generated review content, AIGS is more likely to trigger careful scrutiny and reduce users’ trust and willingness to use it. Therefore, once inconsistency arises between AIGS and user review information, consumers are more likely to reduce their adoption intention toward AIGS as a decision-making basis (Yu et al., 2026). Based on this reasoning, this study proposes the following hypothesis:

**H1.**  *Compared with the consistency condition, AIGS-AUGR inconsistency significantly reduces consumers’ adoption intention toward AIGS.*

### 3.2. The Mediating Role of Perceived Diagnosticity

Within the overarching cue-utilization framework, accessibility–diagnosticity theory explains how cross-cue inconsistency affects consumers’ assessments of the decision-support value of AIGS. Perceived diagnosticity refers to the extent to which a piece of information helps consumers identify product differences, understand product attributes, and support judgment and choice (Z. Jiang & Benbasat, 2007b). Recent studies have shown that diagnosticity remains an important factor influencing information use in online review contexts. According to accessibility–diagnosticity theory, accessible information is more likely to influence judgment when consumers perceive it as sufficiently diagnostic. Consistent with this logic, reviews with greater diagnosticity are more likely to be perceived as helpful (Chen et al., 2024). By contrast, information with insufficient diagnosticity may fail to enter consumers’ subsequent judgment process, even when it is noticed by users.

In the context of AIGS, the importance of perceived diagnosticity is not weakened; rather, it becomes more salient (M. Jia et al., 2026). A study examining AI-generated overviews of customer reviews found that such overviews can enhance consumers’ perceived usefulness of review sections, with perceived diagnosticity serving as a key underlying mechanism (Bian & Che, 2025). In other words, consumers perceive AI-generated overview as valuable because it helps them grasp key information in reviews more quickly and improves the efficiency of their overall judgment of the review section. Consistent with this view, related research further showed that users’ willingness to adopt AIGS depends not only on time savings and interface convenience but also on whether AIGS helps consumers understand review content and make decisions (M. Jia et al., 2026).

From this perspective, whether AIGS is consistent with AUGR directly affects consumers’ judgments of its diagnostic value (Bian & Che, 2025; L. Luo et al., 2026). When the summary is consistent with the rating or the overall tendency of user reviews, consumers are more likely to regard it as an effective condensation of the original evaluations. They may therefore believe that the summary helps them identify product strengths and weaknesses and form a higher perception of usefulness (M. Jia et al., 2026; Jin et al., 2023). By contrast, when AIGS is inconsistent with evaluative cues such as AUGR, consumers are more likely to question whether the summary truly reflects the key points of the reviews, thereby weakening their perceived diagnosticity of the summary (L. Luo et al., 2026). In addition, traditional review research has also found that rating inconsistency reduces review helpfulness, suggesting that when evaluative cues conflict with one another, consumers tend to perceive the information as less useful for forming a clear judgment (Y. Liu et al., 2025a).

Perceived diagnosticity is influenced not only by AIGS-AUGR consistency/inconsistency but is also directly related to consumers’ willingness to use review information (Bian & Che, 2025; Chen et al., 2024). Prior research has shown that perceived diagnosticity is an important antecedent of review adoption (Bao & Zhu, 2023). Therefore, if AIGS-AUGR inconsistency weakens consumers’ judgments of the diagnostic value of the summary, they are more likely to reduce their intention to adopt it. Based on this reasoning, this study proposes the following hypotheses:

**H2.**  *Compared with the consistency condition, AIGS-AUGR inconsistency significantly reduces consumers’ perceived diagnosticity.*

**H3.**  *Perceived diagnosticity mediates the effect of AIGS-AUGR consistency/inconsistency on adoption intention toward AIGS.*

### 3.3. The Mediating Role of Perceived Authenticity

The second explanatory pathway concerns consumers’ authenticity attributions. Within the overarching cue-utilization framework, consumers infer whether AIGS faithfully represents the user-generated evaluative cues from which it is derived. Perceived authenticity refers to consumers’ judgment of whether information truly reflects original opinions, has natural expressive features, and remains faithful to real experiences (Fourkan et al., 2026). In the context of GenAI, such judgments are particularly important because consumers may attribute perceived discrepancies to the way AI-generated content has selected, transformed, or represented the underlying information. Research on consumers’ responses to AIGC indicates that consumers tend to approach such content with greater caution (Sahebi et al., 2026). Evidence from branding contexts further suggests that content created using GenAI may weaken audiences’ perceptions of brand authenticity (Brüns & Meißner, 2024). Related empirical findings show that AIGC can reduce perceived brand authenticity and subsequently influence audiences’ behavioral intentions (Ali et al., 2025). Additional research demonstrates that when consumers attribute content creation to AI rather than to humans, their authenticity evaluations decline, thereby weakening favorable behavioral responses (Kirk & Givi, 2025).

In the context of online reviews, authenticity is particularly important. One key reason why online reviews have decision-making value is that consumers regard them as expressions of genuine user experiences (Cheng et al., 2025). When AI is embedded in review systems, perceived authenticity can serve as an important mediating mechanism linking AI-related cues to consumer trust (Drozd & Söilen, 2026). Research on AIGS indicates that users attend to the fairness, authenticity, and effectiveness of the process through which the summaries are generated (M. Jia et al., 2026). Related evidence further suggests that users’ evaluations of AIGS are shaped by their perceptions of the content source (Yhee & Koo, 2026). Comparative research on AI-generated and human-generated reviews further shows that users actively distinguish between the two types of content and adjust their adoption judgments accordingly (Cheng et al., 2025).

From this perspective, the consistency between AIGS and user review information directly shapes consumers’ judgments of its authenticity. When a summary is consistent with AUGR or the overall tendency of user reviews, consumers are more likely to regard it as an authentic representation of user opinions. By contrast, when the summary is inconsistent with the rating, consumers may suspect that the underlying reviews have been selectively summarized or presented in a biased manner, thereby reducing the perceived authenticity of the summary. Empirical evidence indicates that AIGS-AUGR inconsistency weakens users’ attitudes toward AIGS (L. Luo et al., 2026). Related research has also documented systematic linguistic differences between AI-generated and genuine reviews, suggesting that authenticity-related cues may influence users’ acceptance of AI-generated evaluative information (Zhao et al., 2025).

Therefore, AIGS-AUGR inconsistency not only affects consumers’ overall attitudes toward AIGS, but also further reduces their adoption intention by weakening perceived authenticity. Based on this reasoning, this study proposes the following hypotheses:

**H4.**  *Compared with the consistency condition, AIGS-AUGR inconsistency significantly reduces consumers’ perceived authenticity.*

**H5.**  *Perceived authenticity mediates the effect of AIGS-AUGR consistency/inconsistency on adoption intention toward AIGS.*

### 3.4. The Moderating Role of Review Dispersion

Although cross-cue inconsistency may activate both diagnosticity and authenticity concerns, the relative salience of these concerns depends on the structure of the original review environment. Importantly, review dispersion is conceptualized in this study as a boundary condition that changes how consumers interpret cross-cue inconsistency, rather than as a direct determinant of adoption intention. Therefore, its moderating role is expected to occur primarily through the psychological evaluation processes underlying AIGS adoption. Review dispersion captures an important feature of this informational context. Review dispersion refers to the extent to which evaluations in original reviews of the same product diverge, indicating whether reviewers’ opinions are concentrated or clearly divided. Existing research has shown that review dispersion shapes how consumers interpret and use review sets rather than functioning merely as a background characteristic. Empirical evidence indicates that review dispersion influences consumers’ purchase intention through perceived credibility (F. Liu et al., 2023). Related research further suggests that rating dispersion influences consumers’ trust in average ratings and their motivation to consult individual reviews (S. Lee et al., 2021). Research on cross-review incoherence further shows that disagreement within a review set over specific product attributes can alter consumers’ assessments of the overall helpfulness and credibility of that review set (Yin et al., 2023).

When review dispersion is low, original reviews tend to exhibit a strong consensus, and consumers are more likely to form a stable overall impression based on average ratings and a limited number of reviews (Yin et al., 2026). Research has shown that consumers do not rely solely on average ratings; they also use rating distributions to judge whether the evaluative environment is stable. Different types of review variance may have different effects on market demand (N. Lee et al., 2022). Other studies have found that review variance interacts with regulatory focus and product type to influence consumers’ product attitudes, suggesting that the distributional structure of reviews changes how users interpret evaluative information (Yi & Park, 2026). Therefore, under low review dispersion, if AIGS is inconsistent with AUGR or the overall tendency of user reviews, consumers are more likely to interpret such inconsistency as a deviation from an existing consensus rather than as a reasonable summary of diverse opinions. Research on review set inconsistency has also shown that internal inconsistency and external inconsistency can change consumers’ judgments of review set helpfulness and sales outcomes (P. Jiang & Fan, 2026). Accordingly, it can be inferred that under low review dispersion, AIGS-AUGR inconsistency is more likely to damage consumers’ judgments of whether the AIGS authentically reflects user reviews.

Under high review dispersion, the original reviews themselves contain clear disagreements, making it more difficult for consumers to form a definite judgment based on average ratings or individual reviews (Yang et al., 2025). In this situation, the value of AIGS lies not only in compressing information, but also in helping consumers organize dispersed opinions and identify major points of controversy. Studies have found that review inconsistency reduces review helpfulness, whereas rating inconsistency may have different effects, suggesting that consumers distinguish among different levels of inconsistency and adjust their information use accordingly (D. Wang et al., 2025). Some research also indicates that rating–text inconsistency may sometimes encourage consumers to engage in deeper information processing rather than simply rejecting the value of the information (Kwon et al., 2025). In tourism and restaurant review contexts, electronic word-of-mouth (eWOM) dispersion can also influence consumers’ curiosity and purchase intention, indicating that opinion divergence changes how consumers understand the meaning of information (H. Sun et al., 2025). In addition, sentiment quantity, dispersion, and dissimilarity in online reviews jointly influence users’ subsequent responses (Y. Wang et al., 2024). Therefore, under high review dispersion, if the AIGS remains inconsistent with the overall evaluative signal, consumers are more likely to believe that it has failed to effectively organize complex reviews, thereby leading to a stronger decline in perceived diagnosticity.

Taken together, review dispersion is expected to shape the two psychological mechanisms asymmetrically rather than uniformly. High review dispersion increases consumers’ need for AIGS to organize conflicting evaluations, making inconsistency more damaging to perceived diagnosticity, whereas low review dispersion provides a clearer consensus benchmark, making the same inconsistency more damaging to perceived authenticity. Based on this reasoning, this study proposes the following hypotheses:

**H6.**  *Review dispersion moderates the first-stage effect of AIGS-AUGR inconsistency on perceived diagnosticity. The negative effect of cross-cue inconsistency on perceived diagnosticity is stronger under high review dispersion than under low review dispersion.*

**H7.**  *Review dispersion moderates the first-stage effect of AIGS-AUGR inconsistency on perceived authenticity. The negative effect of cross-cue inconsistency on perceived authenticity is stronger under low review dispersion than under high review dispersion.*

**Figure 1:**
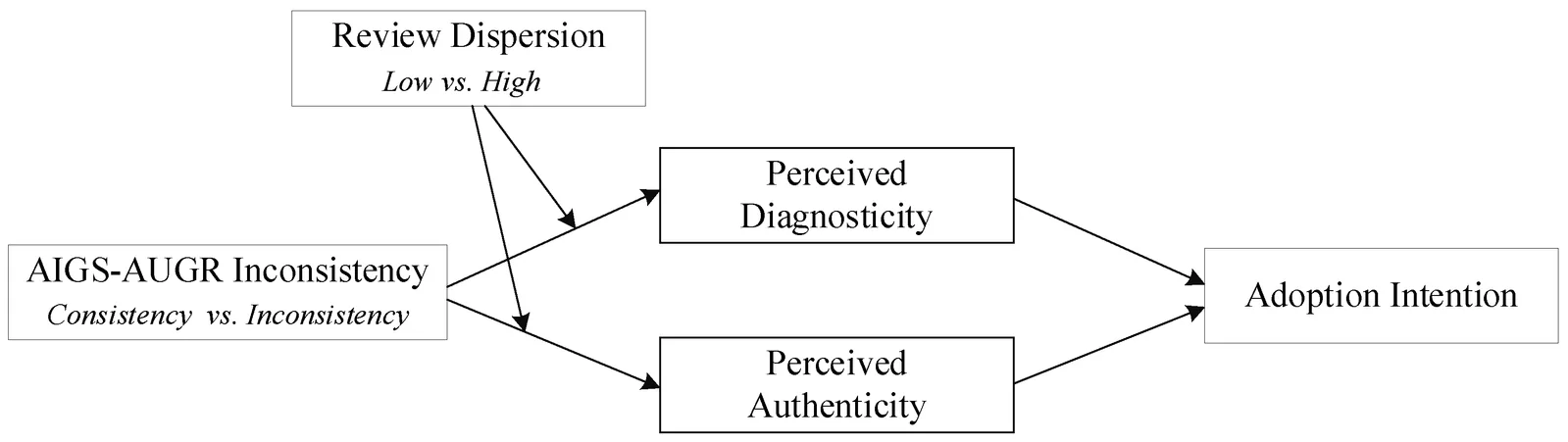
The theoretical model of this study.

Based on these hypotheses, this study constructs the theoretical model shown in Figure 1. Because review dispersion is proposed to influence the psychological mechanisms through which cross-cue inconsistency affects adoption intention, the subsequent analyses further examine whether the indirect effects through perceived diagnosticity and perceived authenticity vary across different levels of review dispersion.

## 4. Methodology

### 4.1. Stimulus Materials

This study employed a scenario-based experimental design. The experimental stimulus consisted of a simulated product review page on an e-commerce platform. Scenario-based experiments are commonly used in research on online reviews and consumer judgment because they allow researchers to manipulate key informational cues while maintaining a relatively realistic decision context (Nakayama & Wan, 2021). Bluetooth headphones were selected as the focal product because they are familiar to consumers, combine characteristics of both search and experience goods, and possess readily evaluable attributes, including connection stability, sound quality, battery life, and wearing comfort (Ma et al., 2026; J. Wang et al., 2025). These characteristics made Bluetooth headphones suitable for constructing both AI-generated review summaries and original review materials.

Each stimulus page presented the product name, a brief product description, the number of reviews, an aggregated user-generated rating (AUGR), an AI-generated review summary (AIGS), and several original user reviews. The experiment adopted a 2 (AUGR level: high vs. low) × 2 (AIGS valence: positive vs. negative) × 2 (review dispersion: low vs. high) between-subjects design, resulting in eight experimental conditions.

Cross-cue consistency was operationalized as the alignment between the level of AUGR and the overall valence of the AIGS. Specifically, a high AUGR paired with a positive AIGS and a low AUGR paired with a negative AIGS constituted the consistency conditions; a high AUGR paired with a negative AIGS and a low AUGR paired with a positive AIGS constituted the inconsistency conditions. This manipulation captured an overall evaluative mismatch between the aggregated rating and the review summary, rather than the inclusion of isolated positive or negative product details within an otherwise directionally aligned summary.

Review dispersion was manipulated through the extent to which the original reviews converged or diverged in their evaluations of the same core product attributes. In the low-dispersion condition, evaluations of the core attributes were generally concentrated in the same direction, although the reviews were not required to agree on every attribute. In the high-dispersion condition, the reviews presented clearly opposing evaluations of the same core attributes. Across the two dispersion conditions, the review materials were designed to be comparable in the number of reviews, approximate text length, coverage of product attributes, and overall amount of information, so that the primary difference concerned the degree of evaluative divergence.

### 4.2. Pilot Study

A pilot study was conducted before the main experiment to assess whether the three experimental factors were perceived as intended. A total of 80 participants were recruited through the Credamo platform and randomly assigned to eight experimental conditions. The pilot study used the same 2 (AUGR: high vs. low) × 2 (AIGS: positive vs. negative) × 2 (review dispersion: low vs. high) between-subjects design described above. After viewing the corresponding simulated e-commerce review page, participants completed the manipulation check items. These items assessed whether participants perceived the intended differences in AUGR level, AIGS valence, and review dispersion.

Specifically, perceived AUGR level was assessed using three items: “I think the overall rating of this product is high”, “I think this product has received good user ratings”, and “Based on the rating, the overall evaluation of this product is positive”. Perceived AIGS valence was assessed using three items: “I think this AI-generated review summary is generally positive”, “This AI-generated review summary mainly emphasizes the product’s advantages”, and “This AI-generated review summary conveys positive user evaluations”. Perceived review dispersion was assessed using four items: “I think user opinions about this product are relatively dispersed”, “Users have considerable disagreement in their evaluations of this product”, “These user reviews do not form a consistent evaluative tendency”, and “There are clear differences among the user reviews”. All items were measured on a seven-point Likert scale, with 1 indicating “strongly disagree” and 7 indicating “strongly agree”. The items corresponding to each manipulation were averaged to form the respective manipulation-check score.

Three independent-samples *t*-tests were conducted to compare the two levels of each manipulated factor. The results showed that participants in the high-AUGR condition reported significantly higher perceived AUGR than those in the low-AUGR condition (M__High rating_ = 6.083, M__Low rating_ = 2.242, t = 31.602, *p* < 0.001). Participants in the positive AIGS condition reported significantly higher perceived positivity of the summary than those in the negative AIGS condition (M__Positive_ = 5.117, M__Negative_ = 2.658, t = 22.468, *p* < 0.001). In addition, participants in the high-dispersion condition reported significantly higher perceived review dispersion than those in the low-dispersion condition (M__High dispersion_ = 5.317, M__Low dispersion_ = 2.525, t = 18.330, *p* < 0.001). These results indicate that the experimental materials successfully manipulated the three key factors in the intended directions, supporting their use in the main experiment.

### 4.3. Participants and Procedure

The main experiment used the same stimulus materials and manipulation procedures as the pilot study. A total of 400 registered Credamo participants from mainland China were recruited. Eligible participants were required to have prior online-shopping experience, be familiar with the role of user reviews in online shopping, and possess a basic understanding of generative artificial intelligence. Participants who met these criteria were randomly assigned to one of the eight experimental conditions.

Before beginning the experiment, participants received information about the study procedures and provided electronic informed consent. They were informed that participation was voluntary and that the experiment involved no foreseeable ethical or privacy risks. Participants were then instructed to imagine that they were considering purchasing a pair of Bluetooth headphones on an e-commerce platform. After viewing one of the eight simulated product review pages, they completed the manipulation-check items, the focal measures, and the demographic questions. Upon completion of the experiment, participants received a modest monetary payment through the Credamo platform.

Responses were excluded when participants exhibited nearly invariant response patterns across questionnaire items, answered the scenario-comprehension items incorrectly, failed the screening items, or completed the questionnaire in an implausibly short period of time. Based on these criteria, 29 responses were excluded, leaving 371 valid responses and yielding a valid-response rate of 92.75%. The eight experimental cells contained between 45 and 48 participants each.

The descriptive statistics of the sample are presented in Table 1. The gender distribution was relatively balanced, with 177 male participants, accounting for 47.71%, and 194 female participants, accounting for 52.29%. In terms of age, the sample was mainly composed of participants aged 18–25 and 26–35, accounting for 54.72% and 33.42%, respectively, suggesting that the participants generally matched the main user characteristics of e-commerce platforms and AI tools. Regarding online shopping frequency, participants who shopped online 1–3 times per week and 4 or more times per week together accounted for 89.75%, indicating a high level of online shopping activity. In terms of AI usage experience, participants who used AI occasionally or frequently accounted for 82.75%, suggesting that most participants had some experience with AI tools. Regarding reliance on online reviews, participants who often read or completely relied on reviews accounted for 76.28%, indicating a relatively high dependence on online reviews. Therefore, the sample was appropriate for the research context.

### 4.4. Measures

All focal constructs were measured using multi-item scales adapted from established studies and contextualized to the setting of AI-generated review summaries. Participants completed the measures after viewing the assigned simulated review page. Unless otherwise specified, all items were assessed on a seven-point Likert scale ranging from 1 (“strongly disagree”) to 7 (“strongly agree”).

The perceived diagnosticity scale was adapted from Z. Jiang and Benbasat (2007a) and Filieri (2015), with minor wording modifications to reflect the context of AIGS. The scale assessed the extent to which the AIGS helped participants understand the review information and evaluate the focal product. The scale included four items: “The AI-generated review summary helps me judge the advantages and disadvantages of this product”, “The AI-generated review summary helps me understand users’ evaluations of this product”, “The AI-generated review summary helps me become familiar with the actual performance of this product”, and “The AI-generated review summary provides valuable information for my product judgment”.

The perceived authenticity scale was adapted from studies on online review authenticity and the authenticity of AIGC, including Evans et al. (2021), Lie et al. (2024), and Bui et al. (2024), with the wording adapted to assess whether the AIGS was perceived as faithfully representing the displayed user reviews. The scale included four items: “The AI-generated review summary authentically reflects user evaluations”, “The AI-generated review summary faithfully summarizes the original review content”, “The AI-generated review summary gives me the impression that it summarizes real user opinions”, and “The AI-generated review summary shows no obvious bias or distortion”. The final item captured participants’ subjective perception of selective emphasis or distortion based on the information presented on the review page; it was not intended to assess the objective presence of algorithmic bias. Conceptually, this item was intended to capture one aspect of representational faithfulness by assessing whether the summary appeared to selectively emphasize or distort the displayed user opinions, rather than to restate the degree of AIGS–AUGR agreement.

The adoption intention scale was adapted from the information adoption model proposed by Sussman and Siegal (2003), as well as studies on online review adoption by C. M. K. Cheung et al. (2008) and G. Jiang et al. (2021). The scale assessed participants’ willingness to use the AIGS as a basis for product evaluation. The scale included four items: “I am willing to refer to the AI-generated review summary when evaluating this product”, “I am willing to regard the AI-generated review summary as an important basis for evaluating this product”, “If I continue to learn about this product, I will use this AI-generated review summary”, and “In similar shopping contexts, I am willing to adopt the AI-generated review summary provided by the platform”.

### 4.5. Analytical Strategy

To preserve the factorial structure of the experiment, the primary analyses were conducted using the original 2 × 2 × 2 between-subjects design. AUGR level (high vs. low), AIGS valence (positive vs. negative), and review dispersion (high vs. low) were entered as fixed factors. The eight experimental cells contained between 45 and 48 participants each.

Because no a priori power-based sample-size target was specified before data collection, a sensitivity power analysis was conducted using the final valid sample to evaluate the smallest effect that could be detected by the present design. Using the parameters for a fixed-effects ANOVA testing special main effects and interactions, with a total sample size of 371, eight experimental groups, α = 0.05, statistical power of 0.80, and numerator degrees of freedom of 1, the design could detect an effect of approximately Cohen’s f = 0.146, corresponding to partial η^2^ ≈ 0.021. Because all main effects and interaction effects in the present 2 × 2 × 2 design had one numerator degree of freedom, this sensitivity estimate applied to the three-way interaction as well as to the lower-order effects.

Perceived diagnosticity, perceived authenticity, and adoption intention were calculated as the unrounded mean of their respective four measurement items. For each dependent variable, a full factorial analysis of variance was conducted using Type III sums of squares. The AUGR × AIGS interaction represents the effect of cross-cue consistency/inconsistency, whereas the AUGR × AIGS × review dispersion interaction tests whether review dispersion moderates this effect. Partial eta squared (partial η^2^) was reported as the effect-size measure.

When a three-way interaction was significant, the AUGR × AIGS interaction was examined separately under high and low review dispersion. Planned comparisons further contrasted the two matched configurations (high AUGR–positive AIGS and low AUGR–negative AIGS) with the two mismatched configurations (high AUGR–negative AIGS and low AUGR–positive AIGS). Bonferroni adjustments were applied to follow-up comparisons. All tests were two-tailed with α = 0.05.

## 5. Empirical Results

### 5.1. Main-Study Manipulation Checks

To assess the effectiveness of the manipulations in the main experiment, Independent-samples *t*-tests were conducted using the 371 valid observations. Participants in the high-AUGR condition reported a significantly higher perceived AUGR level than those in the low-AUGR condition (M__High rating_ = 5.506, M__Low rating_ = 2.487, t(369) = 47.959, *p* < 0.001, 95% CI for the mean difference [2.895, 3.143]). Participants in the positive-AIGS condition also reported significantly higher perceived AIGS valence than those in the negative-AIGS condition (M__Positive_ = 5.534, M__Negative_ = 2.495, t(369) = 46.055, *p* < 0.001, 95% CI [2.909, 3.168]). In addition, perceived review dispersion was significantly higher in the high-dispersion condition than in the low-dispersion condition (M__High dispersion_ = 5.560, M__Low dispersion_ = 2.520, t(369) = 44.517, *p* < 0.001, 95% CI [2.906, 3.174]). The manipulation checks in the main experiment were also successful.

### 5.2. Measurement Quality and Common Method Bias

Before testing the hypotheses, the reliability and validity of the measurement model were assessed. The results indicate that all constructs demonstrated satisfactory internal consistency reliability. Specifically, Cronbach’s α values were 0.850 for perceived diagnosticity, 0.865 for perceived authenticity, and 0.839 for adoption intention, all exceeding the recommended threshold of 0.70 (Tavakol & Dennick, 2011). Composite reliability (CR) values were 0.899, 0.908, and 0.893, respectively, further confirming the reliability of the measurement scales.

Convergent validity was evaluated using standardized factor loadings and average variance extracted (AVE). As shown in Table 2, all standardized factor loadings exceeded 0.70, ranging from 0.789 to 0.878 for perceived diagnosticity, from 0.813 to 0.866 for perceived authenticity, and from 0.790 to 0.840 for adoption intention. Prior research commonly uses 0.70 as the threshold for evaluating factor loadings and suggests that standardized factor loadings above 0.70 are desirable (G. W. Cheung et al., 2023). The AVE values were 0.690, 0.712, and 0.675, respectively, all above the recommended threshold of 0.50 (Fornell & Larcker, 1981). These results demonstrate satisfactory convergent validity.

Discriminant validity was examined using the Fornell–Larcker criterion and the heterotrait–monotrait ratio (HTMT). As presented in Table 3, the square roots of AVE values for perceived diagnosticity (0.831), perceived authenticity (0.845), and adoption intention (0.823) were higher than their correlations with other constructs, supporting discriminant validity. In addition, all HTMT values were below the commonly recommended threshold of 0.90, with values ranging from 0.688 to 0.887, providing further evidence that the three constructs are empirically distinct.

To assess the potential influence of common method bias (CMB), both procedural and statistical remedies were considered. Procedurally, several measures were adopted to reduce the possibility of common method bias, including assuring participants of anonymity, informing them that there were no right or wrong answers, and separating the measurement items from the experimental manipulations. In addition, the independent variables in this study were experimentally manipulated rather than measured through self-reported scales, which further reduced concerns regarding common method bias. Statistically, Harman’s single-factor test was conducted by entering all measurement items into an exploratory factor analysis. The results showed that the first factor accounted for 47.25% of the total variance, which is below the commonly used threshold of 50%. Therefore, no serious common method bias was identified in this study.

### 5.3. Full Factorial ANOVA and Direct Effects

#### 5.3.1. Full Factorial ANOVA Results and Direct-Effect Hypotheses

To examine the effects of the experimental manipulations while preserving the original factorial structure, a full 2 × 2 × 2 between-subjects ANOVA was conducted. AUGR level (high vs. low), AIGS valence (positive vs. negative), and review dispersion (high vs. low) were treated as fixed factors. The interaction between AUGR and AIGS captures the effect of AIGS-AUGR consistency/inconsistency, whereas the three-way interaction among AUGR, AIGS, and review dispersion tests whether review dispersion changes this effect. Partial eta squared (partial η^2^) was reported as the effect-size indicator.

All 371 participants provided complete responses for the three focal constructs. Descriptive statistics for the eight experimental conditions are presented in Table 4.

The full factorial ANOVA results are reported in Table 5. For adoption intention, the AUGR × AIGS interaction was significant (F(1,363) = 1238.51, *p* < 0.001, partial η^2^ = 0.773). The cell-mean pattern showed that the two matched rating–summary configurations produced higher adoption intention than the two mismatched configurations. When descriptively averaged across the two levels of review dispersion, adoption intention was higher in the consistency conditions (*M* = 5.314) than in the inconsistency conditions (*M* = 3.520). Thus, H1 was supported. The three-way interaction was also significant (F(1,363) = 6.46, *p* = 0.011, partial η^2^ = 0.017), suggesting that the effect of AIGS-AUGR consistency/inconsistency on adoption intention varied across different levels of review dispersion. However, this three-way interaction was modest in magnitude relative to the much larger AUGR × AIGS interaction (partial η^2^ = 0.773), indicating that review dispersion qualified rather than outweighed the core effect of cross-cue consistency/inconsistency on adoption intention.

For perceived diagnosticity, the AUGR × AIGS interaction was significant (F(1,363) = 816.38, *p* < 0.001, partial η^2^ = 0.692). The matched configurations resulted in higher perceived diagnosticity than the mismatched configurations. Descriptively, the mean perceived diagnosticity was 5.268 in the consistency conditions and 3.539 in the inconsistency conditions. These findings support H2. Review dispersion also showed a significant main effect (F(1,363) = 70.54, *p* < 0.001, partial η^2^ = 0.163). Furthermore, the AUGR × AIGS × review dispersion interaction was significant (F(1,363) = 31.06, *p* < 0.001, partial η^2^ = 0.079), indicating that review dispersion moderated the influence of AIGS-AUGR consistency/inconsistency on perceived diagnosticity.

For perceived authenticity, the AUGR × AIGS interaction was significant (F(1,363) = 1342.55, *p* < 0.001, partial η^2^ = 0.787). Participants reported higher perceived authenticity in the consistency conditions (*M* = 5.323) than in the inconsistency conditions (*M* = 3.455), consistent with the cell-mean pattern observed in the factorial design. Thus, H4 was supported. The three-way interaction was also significant (F(1,363) = 354.09, *p* < 0.001, partial η^2^ = 0.494), suggesting that the effect of cross-cue inconsistency on perceived authenticity depended on review dispersion.

The factorial ANOVA results demonstrate that AIGS-AUGR inconsistency significantly reduces adoption intention, perceived diagnosticity, and perceived authenticity. Moreover, the significant three-way interactions indicate that review dispersion serves as a boundary condition shaping how consumers interpret cross-cue inconsistency, although its moderating effect on adoption intention itself was comparatively modest. The relatively large AUGR × AIGS interaction effects should be interpreted in light of the experimental manipulation, which used clearly differentiated high versus low AUGR levels and positive versus negative AIGS valence to identify directional cross-cue inconsistency. As a result, the cross-cue conflict presented in the experimental stimuli may have been more salient than the subtler or more ambiguous inconsistencies consumers may encounter on actual platforms.

#### 5.3.2. Additional Validation of Consistency/Inconsistency Classification

Although the primary hypothesis tests retained the original factorial structure, the subsequent mediation analyses reparameterized the AUGR × AIGS interaction as a binary consistency/inconsistency variable. Therefore, an additional analysis was conducted to examine whether the two matched configurations and the two mismatched configurations produced comparable responses on the focal variables. This analysis served as a supplementary validation of the classification and did not replace the full factorial ANOVA.

As shown in Table 6, the consistency classification included the high-AUGR–positive-AIGS configuration (G1, *n* = 93) and the low-AUGR–negative-AIGS configuration (G2, *n* = 91). No statistically significant differences were found between G1 and G2 in perceived diagnosticity (F(1,182) = 1.767, *p* = 0.185), perceived authenticity (F(1,182) = 0.001, *p* = 0.987), or adoption intention (F(1,182) = 0.022, *p* = 0.882). Thus, the two matched configurations generated comparable responses across the three focal outcomes.

The inconsistency classification included the high-AUGR–negative-AIGS configuration (G3, *n* = 94) and the low-AUGR–positive-AIGS configuration (G4, *n* = 93). The two configurations did not differ significantly in perceived diagnosticity (F(1,185) = 2.011, *p* = 0.158), perceived authenticity (F(1,185) = 0.856, *p* = 0.356), or adoption intention (F(1,185) = 0.001, *p* = 0.971). These findings indicate that the two mismatched configurations also produced comparable responses.

The supplementary results provide additional support for classifying the two matched configurations as consistency conditions and the two mismatched configurations as inconsistency conditions in the subsequent mediation analyses. Nevertheless, the full 2 × 2 × 2 factorial model remained the primary basis for hypothesis testing.

These results support the use of the binary consistency/inconsistency contrast in the subsequent mediation analyses.

Supplementary one-way ANOVAs were conducted to examine whether the pooled consistency and inconsistency conditions were clearly differentiated across the three focal outcomes. As shown in Table 7, participants in the consistency conditions reported significantly higher adoption intention than those in the inconsistency conditions (M__consistency_ = 5.314, M__inconsistency_ = 3.520, F(1,369) = 1231.420, *p* < 0.001). This pattern indicates that consumers were substantially less willing to use AIGS as a basis for product evaluation when the valence of the summary conflicted with the aggregated rating.

A similar pattern was observed for perceived diagnosticity. Participants in the consistency conditions reported significantly higher perceived diagnosticity than those in the inconsistency conditions (M__consistency_ = 5.268, M__inconsistency_ = 3.539, F(1,369) = 634.235, *p* < 0.001), indicating that cross-cue inconsistency weakened the perceived usefulness of AIGS for understanding product evaluations and supporting judgment.

Perceived authenticity also differed significantly between the two classifications. Participants in the consistency conditions reported higher perceived authenticity than those in the inconsistency conditions (M__consistency_ = 5.323, M__inconsistency_ = 3.455, F(1,369) = 693.896, *p* < 0.001). Thus, when AIGS diverged from AUGR, participants were less likely to regard the summary as a faithful representation of the underlying user evaluations.

### 5.4. Follow-Up Analysis of Moderating Effects

#### 5.4.1. Decomposition of the Three-Way Interactions

Given the significant three-way interactions identified in the factorial ANOVA, follow-up analyses were conducted to clarify how review dispersion shaped consumers’ responses to AIGS-AUGR consistency/inconsistency. Specifically, separate two-way ANOVAs were conducted within high and low review dispersion conditions to examine whether the AUGR × AIGS interaction differed across levels of review dispersion. The results are summarized in Table 8.

For perceived diagnosticity, the AUGR × AIGS interaction was significant under both low review dispersion (F(1,183) = 1147.21, *p* < 0.001, partial η^2^ = 0.862) and high review dispersion (F(1,180) = 3260.37, *p* < 0.001, partial η^2^ = 0.948). The interaction effect was stronger under high review dispersion, indicating that consumers relied more heavily on AIGS to organize review information when original reviews contained more divergent opinions. Therefore, when AIGS conflicted with AUGR under high review dispersion, consumers were more likely to perceive that the summary failed to provide effective decision-support information. These results support H6.

For perceived authenticity, the AUGR × AIGS interaction was significant under both low review dispersion (F(1,183) = 6317.26, *p* < 0.001, partial η^2^ = 0.972) and high review dispersion (F(1,180) = 739.49, *p* < 0.001, partial η^2^ = 0.804). Unlike perceived diagnosticity, the interaction effect was stronger under low review dispersion. When original reviews showed a relatively concentrated evaluative tendency, deviations between AIGS and AUGR were associated with a larger reduction in perceived authenticity. This pattern is consistent with the interpretation that such deviations may be viewed as selective summarization or inaccurate representation of user opinions. These results support H7.

For adoption intention, the AUGR × AIGS interaction was significant under both low review dispersion (F(1,183) = 2810.21, *p* < 0.001, partial η^2^ = 0.939) and high review dispersion (F(1,180) = 1598.63, *p* < 0.001, partial η^2^ = 0.899). This pattern indicates that, despite the statistically significant moderation by review dispersion, consumers remained strongly sensitive to whether AIGS was consistent with AUGR under both dispersion conditions.

The factorial follow-up analyses demonstrate that review dispersion does not uniformly strengthen or weaken the influence of AIGS-AUGR inconsistency. Instead, it changes the dimension along which consumers evaluate inconsistent AIGS. High review dispersion amplifies the adverse effect of inconsistency on perceived diagnosticity, whereas low review dispersion amplifies its adverse effect on perceived authenticity.

#### 5.4.2. Supplementary Interpretation of the Moderation Patterns

To complement the factorial decomposition reported above and facilitate interpretation of the moderation patterns, supplementary simple-effects analyses were conducted using the binary consistency/inconsistency classification validated in Section 5.3.2. The two matched AUGR–AIGS configurations were pooled as the consistency condition, whereas the two mismatched configurations were pooled as the inconsistency condition. Bonferroni-adjusted pairwise comparisons were used to examine differences between high and low review dispersion within each classification.

**Figure 2:**
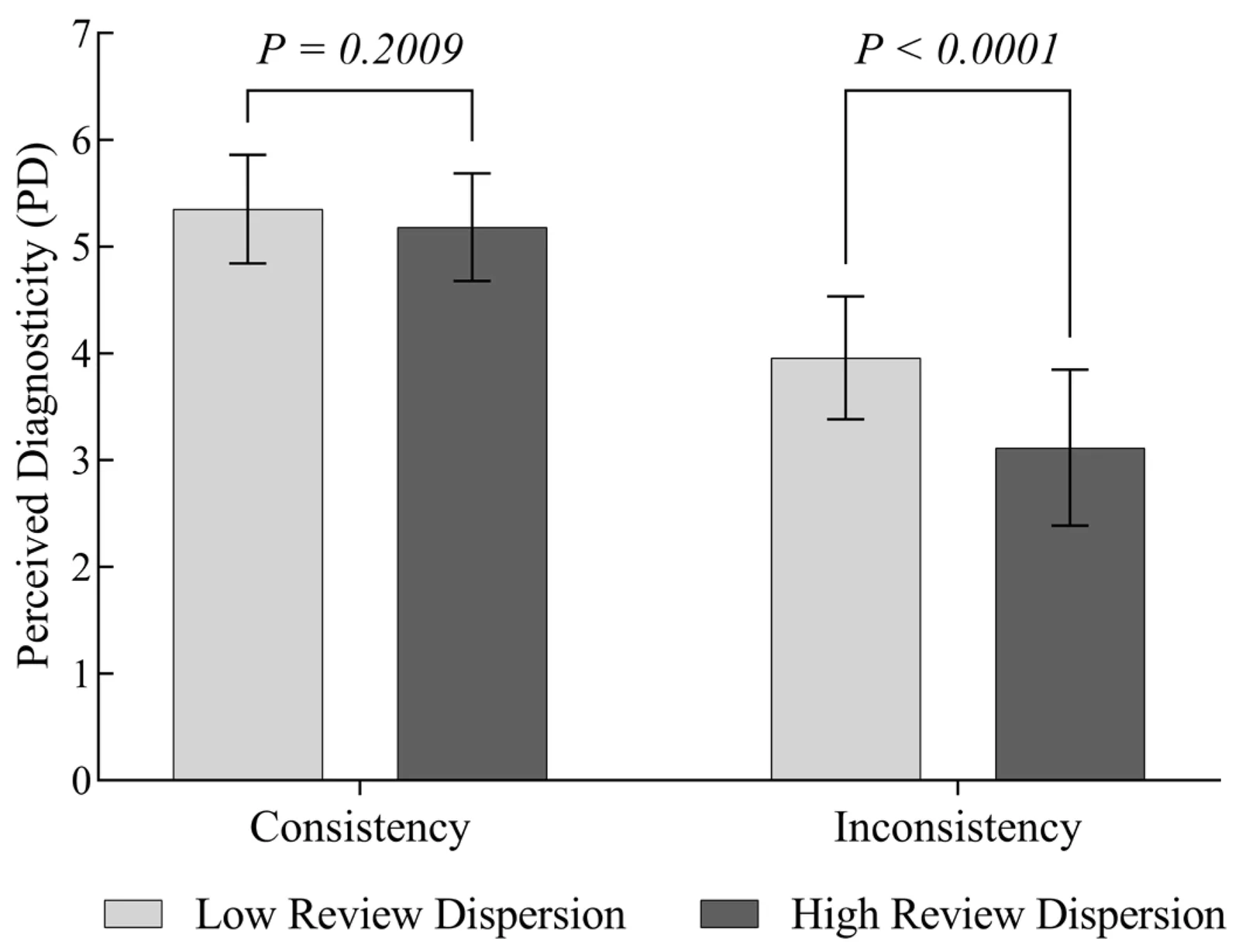
Supplementary Simple Effects Analysis of the Moderating Role of RD on PD.

As shown in Figure 2, review dispersion primarily differentiated perceived diagnosticity under the inconsistency condition. When AIGS was inconsistent with AUGR, perceived diagnosticity was significantly lower under high review dispersion than under low review dispersion (M__Low RD_ = 3.957, M__High RD_ = 3.116, *p* < 0.001). By contrast, when AIGS was consistent with AUGR, perceived diagnosticity did not differ significantly between the high- and low-dispersion conditions (M__Low RD_ = 5.352, M__High RD_ = 5.181, *p* > 0.050). Correspondingly, the descriptive consistency–inconsistency gap was larger under high review dispersion (2.065) than under low review dispersion (1.395). This pattern is consistent with the factorial finding that high review dispersion strengthens the negative effect of AIGS-AUGR inconsistency on perceived diagnosticity.

**Figure 3:**
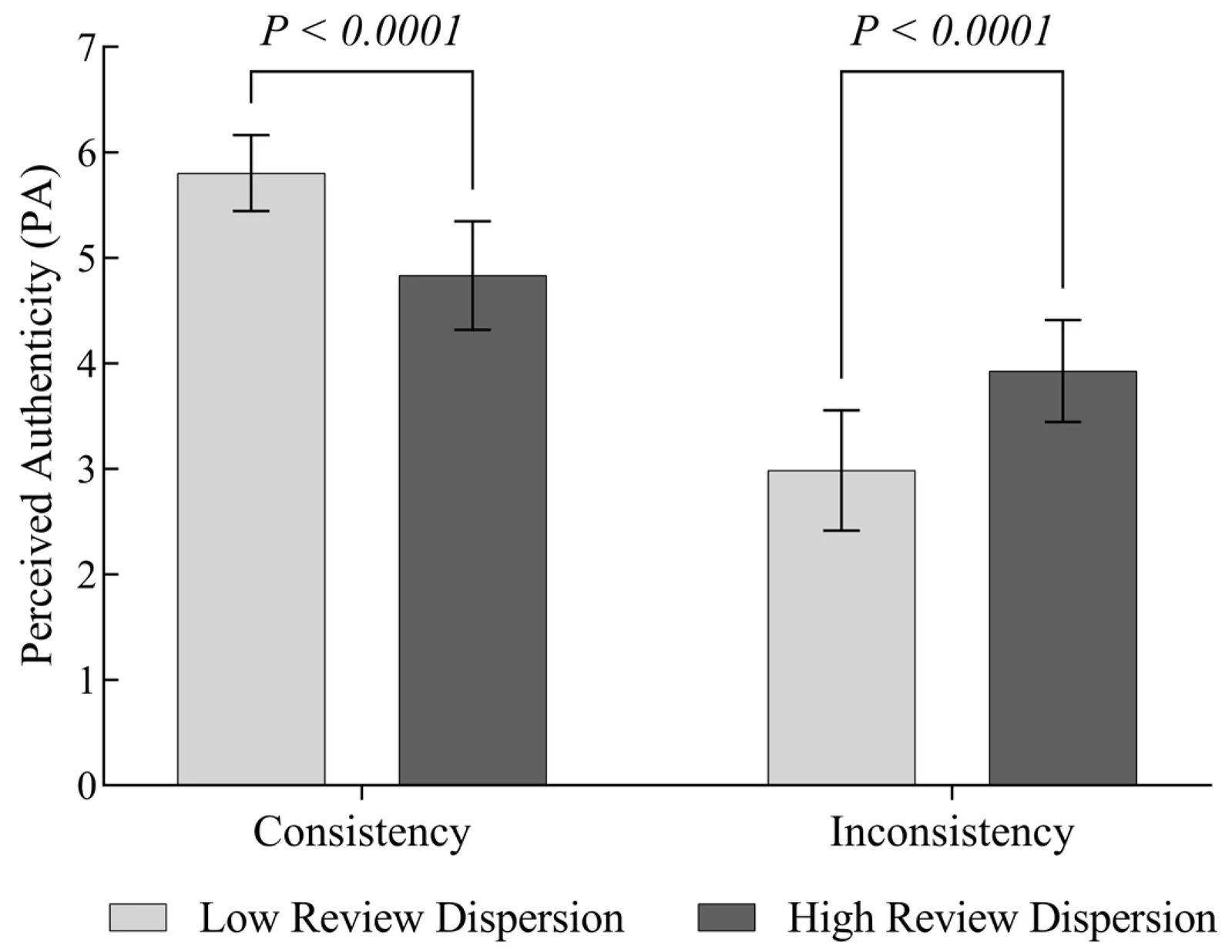
Supplementary Simple Effects Analysis of the Moderating Role of RD on PA.

A different pattern emerged for perceived authenticity. As shown in Figure 3, under the consistency condition, perceived authenticity was significantly higher when review dispersion was low rather than high (M__Low RD_ = 5.804, M__High RD_ = 4.832, *p* < 0.001). Under the inconsistency condition, however, perceived authenticity was significantly lower under low review dispersion than under high review dispersion (M__Low RD_ = 2.987, M__High RD_ = 3.927, *p* < 0.001). Consequently, the descriptive consistency–inconsistency gap was substantially larger under low review dispersion (2.817) than under high review dispersion (0.905). This crossover pattern is consistent with the factorial finding that low review dispersion strengthens the negative effect of AIGS-AUGR inconsistency on perceived authenticity.

### 5.5. Analysis of Mediating Effects

To examine the underlying mechanisms through which AIGS-AUGR inconsistency influences adoption intention, mediation analyses were conducted using PROCESS Model 4 with 5000 bootstrap samples. In the mediation model, AIGS-AUGR inconsistency was coded as the independent variable (0 = consistency, 1 = inconsistency), perceived diagnosticity and perceived authenticity were specified as parallel mediators, and adoption intention was treated as the dependent variable.

As shown in Table 9, AIGS-AUGR inconsistency had a significant negative total effect on adoption intention (β = −1.994, SE = 0.032, *p* < 0.001). After including perceived diagnosticity and perceived authenticity as mediators, the direct effect of inconsistency on adoption intention remained significant (β = −1.452, SE = 0.115, *p* < 0.001), indicating that the two mediators partially explained the negative relationship between cross-cue inconsistency and adoption intention.

Regarding the indirect effects, perceived diagnosticity significantly mediated the relationship between AIGS-AUGR inconsistency and adoption intention (indirect effect = −0.183, 95% CI [−0.343, −0.028]). This finding indicates that inconsistency reduces adoption intention partly because consumers perceive the AI-generated review summary as less useful for identifying product attributes and supporting judgment.

Perceived authenticity also significantly mediated the relationship between AIGS-AUGR inconsistency and adoption intention (indirect effect = −0.361, 95% CI [−0.487, −0.238]). This result suggests that inconsistency decreases adoption intention because consumers become less confident that the AI-generated review summary faithfully represents original user evaluations.

These findings demonstrate that, at the overall unconditional level, AIGS-AUGR inconsistency affects consumers’ adoption intention through two distinct psychological mechanisms: reduced perceived diagnosticity and weakened perceived authenticity. Thus, the parallel mediation analysis supports the proposed dual-pathway mechanism when the indirect effects are estimated without conditioning them on review dispersion.

### 5.6. Moderated Mediation Analysis

To formally test whether review dispersion conditionally influenced the indirect effects of AIGS–AUGR inconsistency on adoption intention, a factorial first-stage moderated mediation analysis was conducted with 5000 bootstrap resamples. Rather than collapsing the four AUGR–AIGS combinations, the analysis retained the original factorial structure. AUGR level and AIGS valence were effect-coded (−0.5 and +0.5), and their interaction was reparameterized as a planned inconsistency contrast (0 = consistency; 1 = inconsistency). Review dispersion was coded as 0 = low and 1 = high. The mediator equations included AUGR, AIGS valence, review dispersion, the inconsistency contrast, the two AUGR/AIGS-by-dispersion interactions, and the inconsistency-by-dispersion interaction. The adoption-intention equation retained the same experimental terms and simultaneously included perceived diagnosticity and perceived authenticity. This model addresses a different inferential question from the unconditional parallel mediation analysis in Section 5.5. Whereas Section 5.5 estimates the overall indirect effects of the binary consistency/inconsistency contrast averaged across review-dispersion conditions, the present model evaluates the mediator-specific indirect effects within the complete factorial structure and tests whether those indirect effects vary across levels of review dispersion. Accordingly, differences between the two analyses should be interpreted as differences between an overall mediation estimate and a more fully specified conditional estimate.

As shown in Table 10 and Table 11, the inconsistency-by-dispersion interaction significantly predicted perceived diagnosticity (B = −0.675, SE = 0.121, t = −5.573, *p* < 0.001). Specifically, the negative indirect effect of inconsistency on adoption intention through perceived diagnosticity was significant under both low review dispersion (indirect effect = −0.200, 95% bootstrap CI [−0.336, −0.075]) and high review dispersion (indirect effect = −0.297, 95% bootstrap CI [−0.497, −0.111]). The index of moderated mediation was significant (Index = −0.097, 95% bootstrap CI [−0.181, −0.032]), indicating that high review dispersion strengthened the negative indirect effect of AIGS–AUGR inconsistency through perceived diagnosticity.

The inconsistency-by-dispersion interaction also significantly predicted perceived authenticity (B = 1.911, SE = 0.102, t = 18.817, *p* < 0.001), indicating that high review dispersion attenuated the negative effect of inconsistency on perceived authenticity. However, after controlling for the complete factorial terms and perceived diagnosticity, perceived authenticity did not significantly predict adoption intention (B = 0.093, SE = 0.052, t = 1.787, *p* = 0.075). Accordingly, the conditional indirect effects through perceived authenticity were not significant under either low review dispersion (indirect effect = −0.261, 95% bootstrap CI [−0.573, 0.029]) or high review dispersion (indirect effect = −0.084, 95% bootstrap CI [−0.186, 0.010]). The corresponding index of moderated mediation was not significant (Index = 0.177, 95% bootstrap CI [−0.020, 0.391]). This result qualifies, rather than contradicts, the significant authenticity-based indirect effect reported in the unconditional mediation model in Section 5.5. The earlier model estimates the overall indirect association through perceived authenticity across review-dispersion conditions, whereas the present model estimates its unique conditional contribution after retaining the full factorial structure. Additional collinearity diagnostics did not indicate serious multicollinearity; therefore, the loss of statistical significance should not be attributed to a multicollinearity artifact. Instead, the results suggest that the authenticity-based indirect effect is sensitive to model specification: it is statistically detectable in the overall mediation model but is not sufficiently robust to remain significant in the more fully specified conditional model.

The results show that review dispersion significantly moderates the first-stage effects of AIGS–AUGR inconsistency on both perceived diagnosticity and perceived authenticity. However, only the diagnosticity-based indirect pathway varies significantly across levels of review dispersion. Thus, review dispersion changes the extent to which inconsistency undermines consumers’ adoption intention through perceived diagnosticity, whereas the evidence does not support a corresponding moderated indirect effect through perceived authenticity. Taken together with the unconditional mediation results, these findings indicate that perceived authenticity contributes to the overall mediation of the inconsistency effect, but its indirect contribution is not statistically robust once the full conditional factorial structure is modeled. Perceived diagnosticity therefore provides the more stable evidence of a conditional indirect mechanism across the two analytical specifications.

## 6. Discussion

### 6.1. Discussion of Research Findings

This study shows that consumers evaluate AIGS within a multi-cue review environment rather than as an isolated source of information. The significant interaction between AUGR and AIGS valence indicates that adoption intention depends on whether the two evaluative cues are aligned. When AIGS was inconsistent with AUGR, consumers reported lower adoption intention than when the two cues were consistent. This finding is consistent with L. Luo et al. (2026), who found that sentiment inconsistency between AIGS and AUGR weakens users’ attitudes toward AI-generated summaries. The present study further shows that the usefulness of AIGS depends not only on its ability to condense large volumes of reviews but also on whether the summary forms a coherent relationship with the user-generated evaluative cues displayed alongside it. Moreover, the direct effect of inconsistency remained significant after perceived diagnosticity and perceived authenticity were included, indicating that the two mechanisms provide a partial rather than complete explanation of the reduction in adoption intention.

The mediation results identify two related but conceptually distinct reasons why cross-cue inconsistency weakens AIGS adoption. AIGS-AUGR inconsistency reduced perceived diagnosticity by making the summary appear less useful for identifying product attributes, organizing review information, and supporting product evaluation. This evidence extends the finding of Bian and Che (2025) that perceived diagnosticity helps explain the usefulness of AI-generated review overviews. At the same time, inconsistency reduced perceived authenticity because the summary appeared less faithful to the user opinions it was expected to represent. This authenticity-based mechanism complements previous evidence that consumers’ adoption of AIGS depends on credibility and helpfulness judgments (M. Jia et al., 2026). At the overall unconditional level, the parallel mediation analysis therefore suggests that inconsistency reduces adoption intention through both impaired decision-support value and weakened representational faithfulness. However, the subsequent conditional analysis qualifies this conclusion: the diagnosticity-based indirect effect remains statistically reliable across the full factorial moderated mediation model, whereas the authenticity-based indirect effect does not. These two pathways explain different aspects of consumers’ responses: perceived diagnosticity concerns what the summary enables consumers to do, whereas perceived authenticity concerns whether the summary adequately represents the underlying reviews.

Review dispersion further changes how consumers interpret AIGS-AUGR inconsistency, but its role differs across the two psychological mechanisms. Under high review dispersion, inconsistency exerted a stronger negative effect on perceived diagnosticity. When original reviews contain sharply divergent evaluations, consumers have a greater need for AIGS to organize conflicting opinions and clarify major points of disagreement. A summary that remains inconsistent with AUGR under these conditions is therefore more likely to be evaluated as ineffective for decision support. Consistent with this interpretation, the negative indirect effect through perceived diagnosticity was stronger under high review dispersion, and the corresponding index of moderated mediation was significant. Under low review dispersion, by contrast, inconsistency produced a stronger reduction in perceived authenticity because the summary departed from a comparatively concentrated evaluative tendency. However, although review dispersion significantly moderated the first-stage effect of inconsistency on perceived authenticity, the conditional indirect effects through perceived authenticity and the corresponding index of moderated mediation were not significant in the integrated factorial model. The findings do not indicate that review dispersion simply shifts the dominant mediation pathway. Instead, they show that review dispersion alters consumers’ initial diagnosticity and authenticity evaluations, while the statistically reliable variation in the indirect effect across dispersion levels occurs through perceived diagnosticity.

### 6.2. Theoretical Contribution

This study contributes to research on GenAI-enabled consumer decision making by repositioning AIGS adoption as a relational judgment problem within a multi-cue review environment. Prior studies have mainly examined consumer responses to AI shopping assistants, AI recommendations, AI advertising, and AI-generated reviews in terms of motivation, trust, disclosure, and source identity (Hermann & Puntoni, 2024; Xie, 2026). For example, GenAI disclosure can reduce consumers’ trust in and attitudes toward service advertisements (Grigsby et al., 2025), while consumers may evaluate online reviews as less useful, credible, and authentic when they believe that the reviews were generated by AI (Amos & Zhang, 2024). Building on but moving beyond this source-based perspective, the present study shows that consumers evaluate AIGS in relation to AUGR and the original reviews displayed within the same interface. Conceptually, AIGS-AUGR inconsistency is therefore not treated as an entirely new form of inconsistency, but as a cross-source and cross-level extension of external inconsistency: a platform-generated summary conflicts with an aggregated user-generated evaluative cue. This positioning connects research on GenAI adoption with established inconsistency typologies and identifies alignment among adjacent evaluative cues as an important condition for understanding consumer responses to AI-generated summaries.

At the mechanism level, the study refines existing explanations of AI-generated information adoption that rely on broad constructs such as usefulness, credibility, or trust (M. Jia et al., 2026; Lei & Liu, 2025; Mun & Hwang, 2024). By distinguishing perceived diagnosticity from perceived authenticity, the study separates the decision-support value of AIGS from its perceived faithfulness to the underlying user evaluations. Perceived diagnosticity captures whether the summary helps consumers identify product strengths and weaknesses, organize review information, and support product judgment, whereas perceived authenticity captures whether the summary is regarded as a faithful and unbiased representation of user opinions. The unconditional parallel mediation analysis shows that both perceived diagnosticity and perceived authenticity contribute to explaining the effect of AIGS-AUGR inconsistency, although the integrated conditional analysis indicates that the authenticity-based indirect effect is less robust once the full factorial structure is taken into account. This pattern clarifies that cross-cue conflict does not merely generate a general loss of trust. Instead, it can impair both the decision-support value and the perceived representational faithfulness of AIGS, while the evidence for a conditional indirect process is stronger for perceived diagnosticity. This distinction also specifies how cue utilization theory operates through the diagnosticity logic of information use and the authenticity-attribution logic of representational fidelity.

The study further extends online review inconsistency research by identifying review dispersion as an informational boundary condition rather than merely a descriptive characteristic of the review distribution. Prior studies have shown that disagreement among ratings, review text, and review content can influence perceived helpfulness and information use, and that different forms of inconsistency may produce different effects across review contexts (Kwon et al., 2025; Y. Liu et al., 2025a; Yin et al., 2023; D. Wang et al., 2025). The present study shows that review dispersion changes the meaning assigned to AIGS-AUGR inconsistency at the first stage of consumer evaluation: high dispersion strengthens the negative effect of inconsistency on perceived diagnosticity, whereas low dispersion strengthens its negative effect on perceived authenticity. Importantly, however, only the diagnosticity-based indirect effect on adoption intention varies significantly across dispersion levels; the corresponding index of moderated mediation through perceived authenticity is not significant. The theoretical contribution therefore does not lie in claiming that review dispersion switches the dominant mediation pathway. Rather, it lies in distinguishing contextual changes in consumers’ initial diagnosticity and authenticity judgments from contextual changes in the complete indirect process leading to adoption intention. This distinction provides a more precise account of when the distributional structure of user reviews shapes the consequences of cross-cue inconsistency.

### 6.3. Managerial Implications

The findings suggest that platforms should manage AIGS through an integrated process of pre-publication validation, dispersion-sensitive presentation, and post-deployment monitoring. These recommendations should be understood as design implications inferred from the experimental findings rather than as platform interventions directly tested in the present study, and their effectiveness therefore requires independent empirical validation. Before an AIGS is displayed, the platform should compare its overall evaluative direction with AUGR and the distribution of the underlying reviews. One practical approach is to place the AUGR signal and the sentiment of the generated summary on a common normalized scale and establish a product-category-specific discrepancy threshold using historical validation data. When the discrepancy exceeds this threshold, the summary could be regenerated, subjected to secondary model or human review, or temporarily withheld from publication. Because the present experiment does not identify a universal numerical cutoff, platforms should calibrate the threshold by balancing missed discrepancies against excessive false warnings. This procedure translates cross-cue consistency from a general design principle into a testable quality-control rule.

The content and presentation of AIGS should also respond to review dispersion and to the different roles of perceived diagnosticity and perceived authenticity. When original reviews are relatively concentrated, the summary should preserve the dominant evaluative direction and indicate the approximate distribution of positive and negative opinions on major product attributes. For example, when a highly rated product receives a limited number of complaints about battery performance, the summary can retain this information while clarifying that it represents a minority of the displayed reviews. Such proportional presentation can reduce the risk that selective emphasis makes the summary appear unfaithful to the underlying evaluations. When reviews are highly dispersed, a single positive or negative conclusion is less useful. In this situation, the summary should organize the principal areas of agreement and disagreement, identify the attributes on which users are divided, and provide links to representative reviews on both sides. A summary might state, for example, that users generally agree on sound quality but report contrasting experiences regarding wearing comfort and battery life. This form of presentation more directly addresses the diagnosticity problem identified under high review dispersion.

If a substantial AIGS–AUGR discrepancy remains after validation, the interface should provide a neutral and informative notice rather than presenting the summary without qualification. For example, the platform could display: “The AI-generated summary and the aggregate rating convey different overall evaluation patterns; please consult the rating distribution and original reviews for additional context”. The warning could also specify the number and time range of reviews used to generate the summary when these differ from the reviews underlying the displayed aggregate rating. After deployment, platforms should evaluate rather than assume the effectiveness of these rules. Alternative discrepancy thresholds and warning formats can be tested through A/B experiments using observable indicators such as AIGS adoption, clicks to original reviews, summary regeneration or correction rates, and user reports of misleading content. Thresholds and presentation rules should then be recalibrated across product categories and over time as new reviews are added. In this way, consistency, diagnosticity, and authenticity become measurable criteria for the ongoing governance of AIGS rather than broad aspirations for platform design.

### 6.4. Limitations and Future Research

Several limitations delimit the scope of the present findings and suggest directions for future research. The first limitation concerns ecological and contextual validity. The scenario-based experiment enabled precise control over AUGR level, AIGS valence, and review dispersion, but it could not fully reproduce consumers’ continuous browsing, repeated comparisons, and exposure to dynamically updated reviews on actual e-commerce platforms. Moreover, the study measured adoption intention rather than consumers’ actual use of AIGS, and the participants were recruited from mainland China through Credamo. Future research could combine field experiments with platform behavioral records, clickstream data, or eye-tracking measures and recruit samples from different cultural and platform contexts to determine whether the observed cross-cue effects persist in more naturalistic settings.

Generalizability across product contexts also remains to be established. Bluetooth headphones were appropriate for the experiment because they are familiar to consumers and contain multiple readily evaluable attributes. Nevertheless, reliance on a single product limits the extent to which the findings can be generalized to other decision contexts. Future studies could compare search-dominant goods, experience-dominant goods, high-involvement or high-risk products, and services to examine whether consumers assign different weights to the diagnosticity and authenticity of AIGS across product categories.

The operationalization of AIGS-AUGR inconsistency represents a further boundary of the study. Inconsistency was defined as a mismatch between the overall valence of AIGS and the direction of AUGR. This manipulation captures directional cross-cue conflict but does not encompass attribute-level discrepancies, temporal misalignment between the reviews summarized and the rating displayed, or cases in which an AIGS adds balanced qualifying information without reversing the overall evaluation. Future research could distinguish contradictory from complementary inconsistency and explainable from unexplainable inconsistency. Future studies could also examine more fine-grained, continuous, or naturally occurring levels of AIGS-AUGR inconsistency to determine whether the observed effects remain stable when cross-cue discrepancies are less salient than those used in the present experiment.

The theoretical model is also deliberately focused in scope. Perceived diagnosticity and perceived authenticity both partially mediated the effect of AIGS-AUGR inconsistency, indicating that additional mechanisms may account for the remaining direct effect on adoption intention. Future research could examine perceived manipulation, platform trust, algorithm aversion, cognitive load, or other responses to cross-cue conflict. In addition, although review dispersion moderated the first-stage effects of inconsistency on both mediators, only the diagnosticity-based indirect effect varied significantly across dispersion levels; the index of moderated mediation through perceived authenticity was not significant. Future studies could therefore investigate whether other characteristics of the review environment—such as review volume, recency, extremity, multimodality, or rating-distribution shape—condition the authenticity-based indirect pathway. Such extensions would clarify whether the asymmetry observed in this study is specific to review dispersion or reflects a broader distinction between consumers’ functional and representational evaluations of AIGS.

## 7. Conclusions

This study examines how cross-cue inconsistency between AI-generated review summaries (AIGS) and aggregated user-generated ratings (AUGR) shapes consumers’ adoption of AIGS in a multi-cue review environment. The findings show that AIGS-AUGR inconsistency significantly reduces adoption intention and that this effect is partially explained by reduced perceived diagnosticity and perceived authenticity. These results indicate that consumers evaluate AIGS not only in terms of its standalone content but also in relation to other evaluative cues presented alongside it.

Review dispersion further shapes how consumers interpret such inconsistency. High review dispersion strengthens the negative effect of inconsistency on perceived diagnosticity, whereas low review dispersion strengthens its negative effect on perceived authenticity. However, only the diagnosticity-based indirect effect varies significantly across review-dispersion conditions. The findings highlight cross-cue consistency, diagnosticity, and authenticity as important considerations for understanding and managing consumers’ adoption of AI-generated review summaries in e-commerce environments.

## Figures and Tables

**Table 1:** Descriptive Statistics of the Sample.

Characteristic	Group	Frequency	Percent
Gender	Male	177	47.71%
	Female	194	52.29%
Age	18–25 years old	203	54.72%
	26–35 years old	124	33.42%
	36–45 years old	32	8.63%
	46 years old and above	12	3.23%
Online shopping frequency	1–3 times per month	38	10.24%
	1–3 times per week	191	51.48%
	4 or more times per week	142	38.27%
AI usage experience	Rarely use	64	17.25%
	Occasionally use	146	39.35%
	Frequently use	161	43.40%
Reliance on online reviews	Almost never read reviews	18	4.85%
	Sometimes read reviews	70	18.87%
	Often read reviews	224	60.38%
	Completely rely on reviews	59	15.90%

**Table 2:** Reliability and Convergent Validity Results.

Variables	Items	Factor Loading	AVE	CR	Cronbach’s α
perceived diagnosticity (PD)	PD1	0.845	0.690	0.899	0.850
	PD2	0.789			
	PD3	0.807			
	PD4	0.878			
perceived authenticity (PA)	PA1	0.852	0.712	0.908	0.865
	PA2	0.813			
	PA3	0.842			
	PA4	0.866			
adoption intention (AdI)	AdI1	0.820	0.675	0.893	0.839
	AdI2	0.836			
	AdI3	0.790			
	AdI4	0.840			

**Table 3:** Discriminant Validity Results.

Fornell–Larcker Criterion				HTMT Results	
Construct	PD	PA	AdI	Construct Pair	HTMT
PD	0.831			PD–PA	0.688
PA	0.591	0.845		PD–AdI	0.874
AdI	0.738	0.755	0.823	PA–AdI	0.887

Note. Diagonal values are the square roots of AVE.

**Table 4:** Cell Means and Standard Deviations by Experimental Condition.

Review Dispersion	AUGR	AIGS	*n*	PD M (SD)	PA M (SD)	AdI M (SD)
High	High	Negative	46	2.97 (0.57)	3.93 (0.37)	3.58 (0.51)
High	High	Positive	46	5.24 (0.54)	4.84 (0.49)	5.24 (0.34)
High	Low	Negative	45	5.12 (0.47)	4.82 (0.54)	5.19 (0.34)
High	Low	Positive	47	3.26 (0.84)	3.92 (0.58)	3.53 (0.64)
Low	High	Negative	48	3.85 (0.65)	2.90 (0.59)	3.46 (0.55)
Low	High	Positive	47	5.39 (0.43)	5.79 (0.35)	5.38 (0.54)
Low	Low	Negative	46	5.31 (0.58)	5.82 (0.37)	5.44 (0.45)
Low	Low	Positive	46	4.07 (0.47)	3.08 (0.54)	3.51 (0.46)

**Table 5:** Full 2 × 2 × 2 Factorial ANOVA Results.

Dependent Variable	Effect	F(1,363)	*p*	Partial η^2^
Adoption intention	AUGR	0.01	0.918	0.000
	AIGS	0.01	0.942	0.000
	RD	1.51	0.219	0.004
	AUGR × AIGS	1238.51	<0.001	0.773
	AUGR × RD	0.95	0.330	0.003
	AIGS × RD	0.01	0.938	0.000
	AUGR × AIGS × RD	6.46	0.011	0.017
Perceived diagnosticity	AUGR	1.57	0.211	0.004
	AIGS	8.48	0.004	0.023
	RD	70.54	<0.001	0.163
	AUGR × AIGS	816.38	<0.001	0.692
	AUGR × RD	0.04	0.839	0.000
	AIGS × RD	0.23	0.634	0.001
	AUGR × AIGS × RD	31.06	<0.001	0.079
Perceived authenticity	AUGR	0.64	0.423	0.002
	AIGS	0.61	0.437	0.002
	RD	0.10	0.748	0.000
	AUGR × AIGS	1342.55	<0.001	0.787
	AUGR × RD	1.31	0.253	0.004
	AIGS × RD	0.52	0.470	0.001
	AUGR × AIGS × RD	354.09	<0.001	0.494

Note. Type III sums of squares were used. AUGR = aggregated user-generated rating, AIGS = AI-generated review summary, RD = review dispersion.

**Table 6:** Mean Comparisons Between Consistency and Inconsistency Conditions.

Classification	Outcome	Configuration 1, M	Configuration 2, M	F (df)	*p*
Consistency	PD	G1: 5.317	G2: 5.217	1.767 (1,182)	0.185
	PA	G1: 5.323	G2: 5.324	0.001 (1,182)	0.987
	AdI	G1: 5.309	G2: 5.319	0.022 (1,182)	0.882
Inconsistency	PD	G3: 3.420	G4: 3.586	2.011 (1,185)	0.158
	PA	G3: 3.407	G4: 3.503	0.856 (1,185)	0.356
	AdI	G3: 3.519	G4: 3.522	0.001 (1,185)	0.971

Note. G1 = high AUGR–positive AIGS (*n* = 93); G2 = low AUGR–negative AIGS (*n* = 91); G3 = high AUGR–negative AIGS (*n* = 94); G4 = low AUGR–positive AIGS (*n* = 93). PD = perceived diagnosticity; PA = perceived authenticity; AdI = adoption intention.

**Table 7:** Supplementary Comparisons Between the Consistency and Inconsistency Conditions.

Variables	Conditions	N	Mean	F	Sig.
Perceived diagnosticity	Consistency	184	5.268	634.235	<0.001
	Inconsistency	187	3.539		
Perceived authenticity	Consistency	184	5.323	693.896	<0.001
	Inconsistency	187	3.455		
Adoption intention	Consistency	184	5.314	1231.420	<0.001
	Inconsistency	187	3.520		

**Table 8:** Simple Interaction Effects of AIGS-AUGR Consistency/Inconsistency under Different Levels of Review Dispersion.

Dependent Variable	Review Dispersion	AUGR × AIGS Interaction	F-Value	*p*-Value	Partial η^2^
Perceived diagnosticity	Low dispersion	Significant	1147.21	<0.001	0.862
	High dispersion	Significant	3260.37	<0.001	0.948
Perceived authenticity	Low dispersion	Significant	6317.26	<0.001	0.972
	High dispersion	Significant	739.49	<0.001	0.804
Adoption intention	Low dispersion	Significant	2810.21	<0.001	0.939
	High dispersion	Significant	1598.63	<0.001	0.899

**Table 9:** Bootstrap Mediation Results.

Effect	β	SE	BootLLCI	BootULCI
Total effect of inconsistency on adoption intention	−1.994	0.032	−2.057	−1.930
Direct effect controlling mediators	−1.452	0.115	−1.679	−1.226
Indirect effect via perceived diagnosticity	−0.183	—	−0.343	−0.028
Indirect effect via perceived authenticity	−0.361	—	−0.487	−0.238
Total indirect effect	−0.544	—	−0.764	−0.330

**Table 10:** Regression Results for the Factorial First-Stage Moderated Mediation Model.

Predictor	PD B (SE)	*p*	PA B (SE)	*p*	AdI B (SE)	*p*
AUGR	−0.064 (0.085)	0.456	−0.099 (0.072)	0.168	−0.037 (0.071)	0.605
AIGS	0.147 (0.085)	0.085	0.076 (0.072)	0.287	−0.036 (0.071)	0.614
RD	−0.171 (0.086)	0.047	−0.972 (0.072)	<0.001	−0.078 (0.087)	0.374
IC	−1.392 (0.085)	<0.001	−2.815 (0.072)	<0.001	−1.461 (0.171)	<0.001
AUGR × RD	−0.025 (0.121)	0.839	0.116 (0.102)	0.253	0.092 (0.100)	0.359
AIGS × RD	0.058 (0.121)	0.634	−0.073 (0.102)	0.470	0.006 (0.100)	0.949
IC × RD	−0.675 (0.121)	<0.001	1.911 (0.102)	<.001	0.179 (0.145)	0.217
PD	—	—	—	—	0.144 (0.043)	0.001
PA	—	—	—	—	0.093 (0.052)	0.075
R^2^	0.719	—	0.825	—	0.784	—
Adjusted R^2^	0.713	—	0.822	—	0.778	—

Note. N = 371. Unstandardized coefficients are reported, with standard errors in parentheses. AUGR and AIGS valence were effect-coded. Review dispersion was coded as 0 = low and 1 = high. The inconsistency contrast (IC) was coded as 0 = consistency and 1 = inconsistency and represents a reparameterization of the AUGR × AIGS interaction.

**Table 11:** Conditional Indirect Effects and Indices of Moderated Mediation.

Mediating Pathway and Review-Dispersion Condition	Effect	Boot SE	BootLLCI	BootULCI
Inconsistency → PD → AdI, low dispersion	−0.200	0.066	−0.336	−0.075
Inconsistency → PD → AdI, high dispersion	−0.297	0.098	−0.497	−0.111
Index of moderated mediation via PD	−0.097	0.037	−0.181	−0.032
Inconsistency → PA → AdI, low dispersion	−0.261	0.153	−0.573	0.029
Inconsistency → PA → AdI, high dispersion	−0.084	0.050	−0.186	0.010
Index of moderated mediation via PA	0.177	0.105	−0.020	0.391
Total indirect effect, low dispersion	−0.460	0.165	−0.795	−0.153
Total indirect effect, high dispersion	−0.380	0.110	−0.607	−0.173
Index of moderated total indirect effect	0.080	0.111	−0.130	0.302

Note. Bootstrap confidence intervals were based on 5000 resamples. A conditional indirect effect or an index of moderated mediation is statistically significant when its 95% bootstrap confidence interval does not include zero. BootLLCI and BootULCI denote the lower and upper limits of the 95% bootstrap confidence interval, respectively.

## Data Availability

The data presented in this study are available from the corresponding author upon reasonable request. The data are not publicly available due to privacy and ethical restrictions.

## Abbreviations

The following abbreviations are used in this manuscript:

AI	Artificial Intelligence
GenAI	Generative Artificial Intelligence
AIGC	AI-Generated Content
UGC	User-Generated Content
AIGS	AI-Generated Summary
AUGR	Aggregated User-Generated Rating
PD	Perceived Diagnosticity
PA	Perceived Authenticity
AdI	Adoption Intention
RD	Review Dispersion
IC	Inconsistency Contrast

## References

[B1-behavsci-16-01516] Ali L., Ali F., Abdalla M. J., Alotaibi S. (2025). Beyond the hype: Evaluating the impact of generative AI on brand authenticity, image, and consumer behavior in the restaurant industry. International Journal of Hospitality Management.

[B2-behavsci-16-01516] Alzate M., Arce-Urriza M., Cebollada J. (2024). Is review visibility fostering helpful votes? The role of review rank and review characteristics in the adoption of information. Computers in Human Behavior.

[B3-behavsci-16-01516] Amos C., Zhang L. (2024). Consumer reactions to perceived undisclosed ChatGPT usage in an online review context. Telematics and Informatics.

[B4-behavsci-16-01516] Baek T. H., Kim J., Kim J. H. (2024). Effect of disclosing AI-generated content on prosocial advertising evaluation. International Journal of Advertising.

[B5-behavsci-16-01516] Bao Z., Zhu Y. (2023). Understanding online reviews adoption in social network communities: An extension of the information adoption model. Information Technology & People.

[B6-behavsci-16-01516] Belanche D., Ibáñez-Sánchez S., Jordán P., Matas S. (2025). Customer reactions to generative AI vs. Real images in high-involvement and hedonic services. International Journal of Information Management.

[B7-behavsci-16-01516] Bian Z., Che C. (2025). How AI Overview of customer reviews influences consumer perceptions in E-commerce?. Journal of Theoretical and Applied Electronic Commerce Research.

[B8-behavsci-16-01516] Bigne E., Ruiz C., Perez-Cabañero C., Cuenca A. (2023). Are customer star ratings and sentiments aligned? A deep learning study of the customer service experience in tourism destinations. Service Business.

[B9-behavsci-16-01516] Brüns J. D., Meißner M. (2024). Do you create your content yourself? Using generative artificial intelligence for social media content creation diminishes perceived brand authenticity. Journal of Retailing and Consumer Services.

[B10-behavsci-16-01516] Bui H. T., Filimonau V., Sezerel H. (2024). AI-thenticity: Exploring the effect of perceived authenticity of AI-generated visual content on tourist patronage intentions. Journal of Destination Marketing & Management.

[B11-behavsci-16-01516] Chen K., Tsai C.-F., Hu Y.-H., Hu C.-W. (2024). The effect of review visibility and diagnosticity on review helpfulness—An accessibility-diagnosticity theory perspective. Decision Support Systems.

[B12-behavsci-16-01516] Cheng X., Zeng A., Yang B., Liu Y., Zhang X. (2025). Online reviews generated by generative artificial intelligence versus human: A study of perceived differences and user adoption behavior. Electronic Commerce Research and Applications.

[B13-behavsci-16-01516] Cheung C. M. K., Lee M. K. O., Rabjohn N. (2008). The impact of electronic word-of-mouth: The adoption of online opinions in online customer communities. Internet Research.

[B14-behavsci-16-01516] Cheung G. W., Cooper-Thomas H. D., Lau R. S., Wang L. C. (2023). Reporting reliability, convergent and discriminant validity with structural equation modeling: A review and best-practice recommendations. Asia Pacific Journal of Management.

[B15-behavsci-16-01516] Cho H. S., Sosa M. E., Hasija S. (2022). Reading between the stars: Understanding the effects of online customer reviews on product demand. Manufacturing & Service Operations Management.

[B16-behavsci-16-01516] Choi H. S., Leon S. (2025). When trust cues help helpfulness: Investigating the effect of trust cues on online review helpfulness using big data survey based on the amazon platform. Electronic Commerce Research.

[B17-behavsci-16-01516] Choi J., Yoo S. H., Lee H. (2023). Two faces of review inconsistency: The respective effects of internal and external inconsistencies on job review helpfulness. Computers in Human Behavior.

[B18-behavsci-16-01516] Drozd D., Söilen K. S. (2026). AI labels, perceived authenticity, and consumer trust in user-generated reviews. Journal of Theoretical and Applied Electronic Commerce Research.

[B19-behavsci-16-01516] Evans A. M., Stavrova O., Rosenbusch H. (2021). Expressions of doubt and trust in online user reviews. Computers in Human Behavior.

[B20-behavsci-16-01516] Filieri R. (2015). What makes online reviews helpful? A diagnosticity-adoption framework to explain informational and normative influences in e-WOM. Journal of Business Research.

[B21-behavsci-16-01516] Fornell C., Larcker D. F. (1981). Evaluating structural equation models with unobservable variables and measurement error. Journal of Marketing Research.

[B22-behavsci-16-01516] Fourkan M., Darani M. M., Wiggins J. (2026). Beyond credibility: Expressive authenticity judgments of online reviews. Journal of Business Research.

[B23-behavsci-16-01516] Grigsby J. L., Michelsen M., Zamudio C. (2025). Service ads in the era of generative AI: Disclosures, trust, and intangibility. Journal of Retailing and Consumer Services.

[B24-behavsci-16-01516] Hermann E., Puntoni S. (2024). Artificial intelligence and consumer behavior: From predictive to generative AI. Journal of Business Research.

[B25-behavsci-16-01516] Jabr W., Rahman M. (2022). Online reviews and information overload: The role of selective, parsimonious, and concordant top reviews. MIS Quarterly.

[B26-behavsci-16-01516] Jia M., Zhao Y., Zhang X. (2026). Navigating the perceived credibility and adoption of AI-generated review summaries in online shopping: An affordance perspective. Information Processing & Management.

[B27-behavsci-16-01516] Jia S. J., Chi O. H., Chi C. G. (2025). Unpacking the impact of AI vs. Human-generated review summary on hotel booking intentions. International Journal of Hospitality Management.

[B28-behavsci-16-01516] Jiang G., Liu F., Liu W., Liu S., Chen Y., Xu D. (2021). Effects of information quality on information adoption on social media review platforms: Moderating role of perceived risk. Data Science and Management.

[B29-behavsci-16-01516] Jiang P., Fan P. (2026). The impact of inconsistency between consumer reviews and management responses in review sets on consumer behavior. International Journal of Hospitality Management.

[B30-behavsci-16-01516] Jiang Z., Benbasat I. (2007a). Research note—Investigating the influence of the functional mechanisms of online product presentations. Information Systems Research.

[B31-behavsci-16-01516] Jiang Z., Benbasat I. (2007b). The Effects of Presentation Formats and Task Complexity on Online Consumers’ Product Understanding. MIS Quarterly.

[B32-behavsci-16-01516] Jin J., Wang A., Wang C., Ma Q. (2023). How do consumers perceive and process online overall vs. individual text-based reviews? Behavioral and eye-tracking evidence. Information & Management.

[B33-behavsci-16-01516] Kakaria S., Simonetti A., Bigne E. (2023). Interaction between extrinsic and intrinsic online review cues: Perspectives from cue utilization theory. Electronic Commerce Research.

[B34-behavsci-16-01516] Kirk C. P., Givi J. (2025). The AI-authorship effect: Understanding authenticity, moral disgust, and consumer responses to AI-generated marketing communications. Journal of Business Research.

[B35-behavsci-16-01516] Kübler R. V., Lobschat L., Welke L., van der Meij H. (2024). The effect of review images on review helpfulness: A contingency approach. Journal of Retailing.

[B36-behavsci-16-01516] Kwon B., Lee J., Min J., Kwak C., Choi H. S. (2025). Beyond the stars: The impact of rating-text inconsistency on perceived review usefulness. Asia Pacific Journal of Information Systems.

[B37-behavsci-16-01516] Langan R., Besharat A., Varki S. (2017). The effect of review valence and variance on product evaluations: An examination of intrinsic and extrinsic cues. International Journal of Research in Marketing.

[B38-behavsci-16-01516] Lee G., Kim H.-Y. (2024). Human vs. AI: The battle for authenticity in fashion design and consumer response. Journal of Retailing and Consumer Services.

[B39-behavsci-16-01516] Lee N., Bollinger B., Staelin R. (2022). Vertical versus horizontal variance in online reviews and their impact on demand. Journal of Marketing Research.

[B40-behavsci-16-01516] Lee S., Lee S., Baek H. (2021). Does the dispersion of online review ratings affect review helpfulness?. Computers in Human Behavior.

[B41-behavsci-16-01516] Lei K., Liu Y. (2025). When AI becomes a shopping advisor: A study on the impact of generative AI review on consumer purchase decision. SAGE Open.

[B42-behavsci-16-01516] Li C., Lin Y., Chen R., Chen J. E. (2025). How do users adopt AI-generated content (AIGC)? An exploration of content cues and interactive cues. Technology in Society.

[B43-behavsci-16-01516] Li Z., Li Y., Zhou J., Ye F., Zhan Y. (2026). How AI-generated summaries of reviews are reshaping E-commerce with competing retailers?. IEEE Transactions on Engineering Management.

[B44-behavsci-16-01516] Lie D. S., Sung B., Stankovic M., Septianto F. (2024). How profanity in influences perceived authenticity and perceived helpfulness of online reviews: The moderating role of review subjectivity. Decision Support Systems.

[B45-behavsci-16-01516] Liu F., Wei H., Wang X., Zhu Z., Chen H. A. (2023). The influence of online review dispersion on consumers’ purchase intention: The moderating role of dialectical thinking. Journal of Business Research.

[B46-behavsci-16-01516] Liu J., Tang J., Yang G., Xiao X. (2025). Unmasking review usefulness: How central, peripheral, and product cues shape consumer perception in online travel reviews. Acta Psychologica.

[B47-behavsci-16-01516] Liu Y., Ma L., Dou Y., Zhu Z., Ma L., Liu Z. (2025a). Injecting new insights: How do review sentiment and rating inconsistency shape the helpfulness of airline reviews?. Information Processing & Management.

[B48-behavsci-16-01516] Liu Y., Wang X., Chen Y. (2025b). Does inconsistency between influencer-generated and user-generated content hurt brands? The role of perceived content credibility. Psychology & Marketing.

[B49-behavsci-16-01516] Luo J., Nan G., Li D. (2026). AI-generated fake review detection. Decision Support Systems.

[B50-behavsci-16-01516] Luo L., Liu L., Zheng Y. (2026). When AI summary is inconsistent with aggregated rating: The role of trust, valence, and algorithmic explanation in consumer attitudes. Telematics and Informatics.

[B51-behavsci-16-01516] Luo L., Liu L., Zheng Y., Chen J. (2024). Visual information and appearance: The impact of visual attributes of user-generated photos on review helpfulness. Telematics and Informatics.

[B52-behavsci-16-01516] Ma J., Zhang D., Chen C., Du H. S. (2026). Matching generative AI word-of-mouth with product type: Impact on consumer adoption and trust. Journal of Retailing and Consumer Services.

[B53-behavsci-16-01516] Mardumyan A., Siret I. (2023). When review verification does more harm than good: How certified reviews determine customer–brand relationship quality. Journal of Business Research.

[B54-behavsci-16-01516] Mousavizadeh M., Koohikamali M., Salehan M., Kim D. J. (2020). An investigation of peripheral and central cues of online customer review voting and helpfulness through the lens of elaboration likelihood model. Information Systems Frontiers.

[B55-behavsci-16-01516] Mun I. B., Hwang K.-H. (2024). Understanding ChatGPT continuous usage intention: The role of information quality, information usefulness, and source trust. Information Development.

[B56-behavsci-16-01516] Nakayama M., Wan Y. (2021). A quick bite and instant gratification: A simulated yelp experiment on consumer review information foraging behavior. Information Processing & Management.

[B57-behavsci-16-01516] Park J., Park H. (2025). Understanding the impact of inconsistency on the helpfulness of online reviews. Journal of Theoretical and Applied Electronic Commerce Research.

[B58-behavsci-16-01516] Pocchiari M., Proserpio D., Dover Y. (2025). Online reviews: A literature review and roadmap for future research. International Journal of Research in Marketing.

[B59-behavsci-16-01516] Qiao S., Meng Y., Feng W. (2025). Rich in modality, poor in value? Understanding the impact of multimodality on review helpfulness. Journal of Retailing and Consumer Services.

[B60-behavsci-16-01516] Qiu K., Zhang L. (2024). How online reviews affect purchase intention: A meta-analysis across contextual and cultural factors. Data and Information Management, Systematic Review and Meta-Analysis in Information Management Research—Part II.

[B61-behavsci-16-01516] Rosillo-Díaz E., Muñoz-Rosas J. F., Blanco-Encomienda F. J. (2024). Impact of heuristic–systematic cues on the purchase intention of the electronic commerce consumer through the perception of product quality. Journal of Retailing and Consumer Services.

[B62-behavsci-16-01516] Sahebi S., Formosa P., Bankins S. (2026). The AI penalty and disclosure paradox: Trust, authenticity and knowledge uptake in AI-mediated communication. Computers in Human Behavior: Artificial Humans.

[B63-behavsci-16-01516] Sun H., Qiu M., Feng W. (2025). Impact of electronic word-of-mouth dispersion on tourists’ purchase intentions for local food restaurants. Journal of Hospitality & Tourism Research.

[B64-behavsci-16-01516] Sun S., Sun H., Xu H., Li H., Wang S. (2025). Unlocking the power of multimodal online reviews: A multisensory perspective. Tourism Management.

[B65-behavsci-16-01516] Sun X., Han M., Feng J. (2019). Helpfulness of online reviews: Examining review informativeness and classification thresholds by search products and experience products. Decision Support Systems.

[B66-behavsci-16-01516] Sussman S. W., Siegal W. S. (2003). Informational influence in organizations: An integrated approach to knowledge adoption. Information Systems Research.

[B67-behavsci-16-01516] Tavakol M., Dennick R. (2011). Making sense of Cronbach’s alpha. International Journal of Medical Education.

[B68-behavsci-16-01516] Wan M. S. K., Sam A. F. (2024). From praise to polarization: The role of controversial reviews in shaping consumer behavior in the PC game market. Journal of Marketing Analytics.

[B69-behavsci-16-01516] Wang D., Xia Q., Feng Y., Cheng T. C. E. (2025). Unravelling the effects of two inconsistencies on online review helpfulness: Evidence from TripAdvisor. Decision Support Systems.

[B70-behavsci-16-01516] Wang J., Liu Y., Qiu Z., Zhao Z., Wang M., Lan H. (2025). Shopping for others: How does inconsistency in online reviews affect purchase intentions differently?. Frontiers in Psychology.

[B71-behavsci-16-01516] Wang P., Zhang H., Yuan X., Zhang X. (2025). Beyond the stars: Unpacking the impact of score-textual inconsistency of online reviews on hotel performance. International Journal of Hospitality Management.

[B72-behavsci-16-01516] Wang S., Karmakar S., Wang F., Pei Y. (2025). Content dissimilarity and online review helpfulness: Contextual insights. Journal of Business Research.

[B73-behavsci-16-01516] Wang Y., Ngai E. W. T., Li K. (2024). Effects of sentiment quantity, dispersion, and dissimilarity on online review forwarding behavior: An empirical analysis. Journal of Retailing and Consumer Services.

[B74-behavsci-16-01516] Wang Y., Zhang Z., Wang C., Li Z., Law R., Zhang Z. (2026). Too concise to decide? The impact of AI-generated review summaries on tourist decision satisfaction. Tourism Management.

[B75-behavsci-16-01516] Xie G. (2026). The impact of generative AI shopping assistants on E-commerce consumer motivation and behavior: Consumer-AI interaction design. International Journal of Information Management.

[B76-behavsci-16-01516] Yang J., Duan H., Kong D. (2025). Consistency matters: Impacts of dimension-level characteristics on the helpfulness of multi-dimensional reviews. Decision Support Systems.

[B77-behavsci-16-01516] Yhee Y., Koo C. (2026). Seeing AI as human or machine? Effects of transparency, valence, and readability on review summary helpfulness. Tourism Management.

[B78-behavsci-16-01516] Yi E., Park D.-H. (2026). Understanding the power of diverging voices: Exploring the interplay of regulatory focus, product type, and review variance on product attitude. Information & Management.

[B79-behavsci-16-01516] Yin D., Bond S. D., Zhang H. (2026). The illusion of authenticity in online reviews: Truth bias and the role of valence. Information Systems Research.

[B80-behavsci-16-01516] Yin D., de Vreede T., Steele L. M., de Vreede G.-J. (2023). Decide now or later: Making sense of incoherence across online reviews. Information Systems Research.

[B81-behavsci-16-01516] Yu X., Xu D. J., Li K. (2026). Effects of AI reviews on consumers’ purchase intention: Influence of product category, review breadth, and consumer review volume. Decision Support Systems.

[B82-behavsci-16-01516] Zhai M., Wang X., Zhao X. (2024). The importance of online customer reviews characteristics on remanufactured product sales: Evidence from the mobile phone market on amazon.com. Journal of Retailing and Consumer Services.

[B83-behavsci-16-01516] Zhang R., Yu Z., Yao W. (2024). Navigating the complexities of online opinion formation: An insight into consumer cognitive heuristics. Journal of Retailing and Consumer Services.

[B84-behavsci-16-01516] Zhang Y., Li Q., Yan J. (2025). Exploring multimodal factors in online reviews: A machine learning approach to evaluating content effectiveness. Journal of Retailing and Consumer Services.

[B85-behavsci-16-01516] Zhao Y., Tang S., Zhang H., Lyu L. (2025). AI vs. human: A large-scale analysis of AI-generated fake reviews, human-generated fake reviews and authentic reviews. Journal of Retailing and Consumer Services.

